# Cold storage of mouse hearts prior to cardiomyocyte isolation preserves electromechanical function, microstructure, and gene expression for 24 h

**DOI:** 10.1007/s00395-025-01131-y

**Published:** 2025-07-29

**Authors:** Benedikt Pfeilschifter, Aiora Martinez-Vilchez, Zafar Iqbal, Prapassorn Potue, Dominik J. Fiegle, Karoline Morhenn, Alexander P. Schwoerer, Tilmann Volk, Thomas Seidel

**Affiliations:** 1 Institute of Cellular and Molecular Physiology, Friedrich-Alexander-University Erlangen-Nürnberg, Erlangen, Germany; 2 Department of Cellular and Integrative Physiology, University Medical Centre Hamburg-Eppendorf, Hamburg, Germany

**Keywords:** Reduction of animals, Heart conservation, Ischemia, Excitation–contraction coupling, Gene expression

## Abstract

Isolation of myocytes from mouse hearts, especially of transgenic animals or disease models, is crucial in cardiac research. The presumption that cardiomyocytes must be isolated immediately after heart procurement to avoid deterioration implies that transgenic mouse lines must be present on site, causes schedule inflexibility, and hampers collaborations, thereby increasing the number, suffering, and costs of animals. This study challenges this presumption by investigating whether the cell isolation can be postponed for 24 h without affecting the results. Adult mouse hearts were subjected to enzymatic myocyte isolation immediately after excision (CTRL) or after 24 h of cold storage (CS). Sufficient numbers of viable cardiomyocytes were obtained in all groups. The transverse-axial tubular system was unchanged in CS versus CTRL. No significant changes were detected in cell capacitance, resting membrane potential, action potential shape and duration, amplitudes, and kinetics of the K^+^ currents I_to_, I_K1_, and I_K_. Sarcomere length, contractility, and relaxation as well as Ca^2+^ signals were equivalent in CS and CTRL at pacing rates of 1–4 Hz. Mitochondrial function assays also yielded equivalence. RNA sequencing yielded only 128 differentially expressed genes, which were mainly related to immune cell function and inflammation. Key findings were reproduced in infarcted mouse hearts, which were shipped overnight as a proof of principle. This study demonstrates that the isolation of cardiomyocytes can be postponed up to 24 h after the procurement of the heart. This opens up new possibilities for collaboration between different laboratories, increases experimental flexibility, and allows to reduce the number of experimental animals by avoiding unnecessary propagation of transgenic lines.

**Supplementary Information:**

The online version contains supplementary material available at 10.1007/s00395-025-01131-y.

## Introduction

Cardiomyocytes isolated from adult animal or human hearts remain a fundamental method of basic cardiac research [16, 17, 27, 31, 52]. To isolate primary cardiomyocytes, the heart must be removed and, consequently, the animal is sacrificed. The obtained myocytes are then used for a plethora of immediate live-cell experiments; for instance, electrophysiological measurements and Ca^2+^ imaging, cell culture, gene transfer, or are fixed and stained for microscopic analyses. Although it has been demonstrated that some artifacts, for example structural changes, are introduced during cell isolation or occur quickly thereafter [5], a presumption in nearly all of these experiments is that the functional and structural quality of the cells is widely preserved and therefore remains representative of the in vivo conditions at least for a few hours after isolation. Importantly, such high quality is an essential prerequisite for meaningful experiments [52]. However, it has been shown that cardiac myocytes remodel already within 24 h after isolation when kept in culture. This comprises changes in electrophysiology, excitation–contraction coupling, contractility, cell size, and shape [1, 30], as well as gene expression [36]. Thus, to investigate myocytes that represent the conditions in vivo, it is important to conduct experiments within a few hours after cell isolation. Furthermore, ischemia and hypoxia of the heart may cause irreversible damage of the myocytes within short periods of time even before cell isolation [20, 49]. This knowledge has led most researchers to assume that great haste is warranted to keep the time between heart removal and cell isolation as short as possible [9, 21, 35, 48].

Notably, carrying out cell isolation immediately after removal of the heart or procurement of myocardial samples entails several obstacles and disadvantages. It not only constrains the laboratory workflow but may also lead to long and inconvenient working hours. For these reasons, some experiments must be postponed or even canceled, or important tissue samples, e.g., donated by patients during cardiac surgery, cannot be investigated. Furthermore, inter-laboratory collaborations are hampered, increasing the number of laboratory animals. This becomes clear if we consider an example scenario where a research group in laboratory A kills an animal whose heart or a myocardial sample could be used by another group in laboratory B. A might be interested only in the kidney, leaving the heart completely intact, or A uses only a part of the heart, which is often the case when a large animal, e.g., a pig, is sacrificed. If laboratory B is too far away from A, B concludes that the transport time is too long and rejects the possibility of collaboration. Instead, B will sacrifice their own animals. In the case of transgenic animals, the increase in laboratory animals and costs becomes even more pronounced, because the transgenic line must be established and maintained in laboratory B. Thus, the assumed need to isolate cells immediately after procurement of a heart or myocardial samples increases the overall costs, may delay projects, and increases the number and potential suffering of laboratory animals by replication.

However, recent findings question the assumption that cardiomyocyte isolation must be carried out immediately. On the whole-organ level, it was already shown many years ago that functional preservation of rabbit hearts in a cold storage solution is possible for more than 24 h [45]. Fitting to this finding, myocardial tissue slices from humans and pigs used for long-term culture did not show functional deterioration when prepared after 24 h of cold storage when compared with slices prepared after only a few hours [14, 43]. Another study using rabbit hearts showed that cardiomyocytes isolated from left-ventricular tissue blocks that had been stored for 24 h or 48 h at 4 °C were not different from immediately isolated cells in terms of important parameters like resting membrane potential or sarcomere length [17]. These studies suggest that, contrary to common believe, the structure, function, and gene expression of ventricular cardiac myocytes can be preserved for at least 24 h. Yet, the assessment of cardiomyocytes did not include electrophysiology, Ca^2+^ signals and gene expression. Also, these studies were not carried out in mouse hearts, which are most commonly used because of the low costs and large number of transgenic animals that are available. Furthermore, these studies described an initial perfusion of the hearts immediately after excision. If heart procurement is done by a collaborator or must be carried out within limited time, coronary perfusion could be problematic, especially in the case of a mouse heart where puncturing the small vessels requires special equipment, skill, and experience.

In this study, we describe a simple protocol for heart preservation and show comprehensively if and to what extent a 24 h delay between heart removal and cardiomyocyte isolation affects experimental results of cellular structure, electrophysiology, intracellular Ca^2+^ handling, excitation–contraction coupling, and mRNA expression in left-ventricular cells of adult mice. We also compare heart preservation after initial perfusion with heart preservation without perfusion, the latter of which is much easier and faster to achieve for researchers with no specific experience.

## Materials and methods

### Heart acquisition

All animal procedures conform to the guidelines from Directive 2010/63/EU of the European parliament. The use of mice for this study was approved by the Animal Care and Use Committee Mittelfranken, Bavaria, Germany and the Ministry for Social Affairs, Family, Health and Consumer Protection, Hamburg, Germany. Male and female C57BL/6N mice (10–20 weeks old) were anaesthetized by inhalation of 4% isoflurane and subsequently killed in narcosis by cervical dislocation. Immediately afterward, the thorax was opened with scissors and the heart, including the ascending part of the aorta, quickly removed.

### Heart preparation

#### Storage conditions

After excision, the hearts were assigned to one of three experimental groups. In the control group (CTRL), hearts were immediately subjected to cell isolation. In the second experimental group, we perfused the heart briefly via the aorta with 4° *Cold storage solution*, using a 5 ml syringe to wash out blood of the coronary vessels (group name: cold storage after perfusion, CSP) and then placed it into a 50 ml tube filled with *Cold storage solution*. In the third experimental group, we omitted perfusion and placed the heart immediately into a 50 ml tube filled with *Cold storage solution* (group name: cold storage, CS). Hearts of the CSP and CS groups were then stored in *Cold storage solution* for 24 h in a fridge at 4 °C. In two additional groups, hearts were perfused upon removal accordingly to CSP group, but the storage period before the isolation was extended to 48 h and 72 h (CSP48h and CSP72h).

#### Infarct model

As a proof of principle experiment, C57BL/6 J mouse (12–25 weeks old) hearts were obtained at the institution at the University Medical Centre Hamburg-Eppendorf and shipped to the institution at the Friedrich-Alexander-University Erlangen-Nürnberg, using an overnight courier service. Hearts were obtained from healthy control mice and mice that were subjected to a ultrasound-guided, minimal-invasive surgery creating a myocardial infarction via high-frequency coagulation of the LAD coronary artery, as described elsewhere [44]. In brief, to induce myocardial infarction, mice were anaesthetized by inhalation of 4% isoflurane. Blood in the proximal part of the left anterior descending artery was coagulated by applying high-frequency pulses with an electrode. Thirty minutes prior to the induction surgery and 6–8 h thereafter, mice received a subcutaneous buprenorphine injection of 0.1 mg/kg body weight. In the following days, mice received 200 mg/kg sodium metamizole in sweetened drinking water.

Successful induction of infarcts was confirmed by ST segment elevation in electrocardiography as well as akinesia in echocardiography. Two days after myocardial infarction, mice were anaesthetized by an intraperitoneal injection of pentobarbital (250 mg/kg body weight) and killed by cervical dislocation. Hearts were immediately excised and placed into a 5 ml tube filled with *Cold storage solution* without prior flushing of the coronary arteries. They were shipped from Hamburg to Erlangen overnight while being cooled on ice. Cell isolation took place on the following day as described below. The control group consisted of hearts from mice that did not receive MI surgery and were treated and shipped to Erlangen in the same way as the MI group.

### Solutions

The following solutions were used during the experiments.

#### Homogenization buffer

150 mM NaCL, 50 mM Tris, 1 mM Na_2_EDTA, pH 7.4; immediately before homogenisation, 1 pill of protease inhibitor, and phosphatase Inhibitor (cOmplete, EDTA free, and PhosSTOP, Roche Diagnostics GmBH, Mannheim, Germany) and was added to 50 ml buffer.

#### *Low Ca*^*2*+^*solution*

138 mM NaCl, 4 mM KCl, 1 mM MgCl_2_, 0.33 mM NaH_2_PO_4_, 10 mM glucose, 10 mM HEPES, 4.5 mM CaCl_2_, 5 mM EGTA, pH 7.30 (adjusted with NaOH).

#### *Ca*^*2*+^*free solution*

130 mM NaCl, 5.4 mM KCl, 0.5 mM MgCl_2_, 0.33 mM NaH_2_PO_4_, 22 mM glucose, 25 mM HEPES + 0.1% bovine serum albumin (BSA, Roth, 0163.2), gassed with 5% CO_2_ at 37° for 20 min, pH 7.40 (adjusted with NaOH).

#### Restitution solution

Ca^2+^ free solution + penicillin/streptomycin (1:100, Biochrom, A2213) + 1 mM CaCl_2_.

#### Cold storage solution

138 mM NaCl, 5.4 mM KCl, 2 mM MgCl_2_, 0.33 mM NaH_2_PO_4_, 10 mM glucose, 0.5 mM CaCl_2_, 10 mM HEPES, 30 mM butanedione monoxime (BDM), pH 7.40 (adjusted with NaOH).

#### Patch clamp solution

138 mM NaCl, 4 mM KCl, 1 mM MgCl_2_, 0.33 mM NaH_2_PO_4_, 10 mM glucose,10 mM HEPES, 2 mM CaCl_2_, pH 7.30 (adjusted with NaOH).

#### Pipette solution

120 mM glutamic acid, 10 mM KCl, 4 mM MgCl_2_, 10 mM EGTA, 10 mM HEPES, 2 mM Na_2_ATP, pH 7.20 (adjusted with KOH).

#### *Ca*^*2*+^*imaging solution*

138 mM NaCl, 4 mM KCl, 1 mM MgCl_2_, 0.33 mM NaH_2_PO_4_, 10 mM glucose,10 mM HEPES, 2 mM CaCl_2_, pH 7.40 (adjusted with NaOH).

### Troponin T test

To investigate potential cardiomyocyte degradation during storage, we took samples from the supernatant of the *Cold storage solution* and measured troponin T concentrations with a commercial point-of-care device (cobas h 232, POC Troponin T, Roche Diagnostics GmbH, Mannheim, Germany). To estimate the total troponin T amount in a mouse heart, we homogenized hearts immediately after excision in 4 ml cold *homogenization buffer* with an immersion blender. The total buffer volume was then complemented to 40 ml. Samples were taken and further diluted to a measureable concentration.

### Cell isolation and culture

Cardiomyocytes were enzymatically isolated from the left ventricle via coronary perfusion, using a Langendorff apparatus as described earlier for mouse [51] and rat [40, 50] cardiomyocytes. In the CTRL group, hearts were excised and initially perfused for 5 min via the aorta with *Low Ca*^*2*+^ *solution*. This initial perfusion was followed by perfusion for 16–20 min with a mix of 2.5 U/ml proteinase XIV (Sigma Lot 0000369483) and 374 U/ml collagenase II (Sigma Lot 0000231168) dissolved in *Low Ca*^*2*+^ *solution*. Finally, hearts were perfused for 5 min with *Ca*^*2*+^ *free solution* supplemented with 100 µM Ca^2+^. After the dissection of the left and right ventricles, the left ventricle was cut into pieces and then dissociated by mechanical force and gentle agitation. Remaining pieces were collected and disposed. The cells were then left to rest for 5 min. Subsequently, the extracellular solution, containing 100 µM Ca^2+^, was replaced in two steps. First, 50% of the solution was replaced by *Restitution solution*. Cells were then left to adapt to these conditions (0.55 mM Ca^2+^) for 5–10 min. Second, the entire extracellular solution was replaced by *Restitution solution,* increasing the Ca^2+^ concentration to approximately 1 mM. Note that after rewarming the heart to 35–37 °C, the heart and cells were maintained at this temperature during all steps described, using heating pads or incubators. After another 10–15 min, the cells were resuspended in culture medium until experimental use for 1–8 h, which consisted of M199 containing 1.8 mM Ca^2+^ (Sigma, 4530), supplemented with 0.1% BSA, 100 U/ml penicillin, and 0.1 mg/ml streptomycin. For storage, an incubator with an atmosphere of 37 °C, 85% relative humidity, and 5% CO_2_ was used. This procedure was identical in all three experimental groups (CTRL, CSP, and CS). In some experiments, a fraction of the cardiomyocytes was kept in culture under these conditions in the described M199-based culture medium for up to 24 h.

### Cell counting

The cardiomyocyte yield was determined immediately after cell isolation and after 6 h and 24 h in culture by means of a Neubauer chamber. Cells were suspended in a defined volume of 5 ml, of which 500 µl were removed and concentrated by gentle centrifugation (3 min, 50 g) and subsequent removal of 250 µl supernatant. The cells were resuspended in the remaining medium. Five samples of the suspension were then loaded into a Neubauer chamber and images taken with a camera (Axiocam 208 color, Zeiss, Germany) connected to an inverted light microscope (DM IL, Leica, Germany). Intact cells and cell fragments were counted later by a researcher blinded against the experimental groups. The cell yield of each isolation was identified as the mean value of the five technical replicates.

### Analysis of the transverse-axial tubular system

Cells were stained with Di-8 ANEPPS and transferred to a chamber with a coverslip bottom on a Zeiss inverted confocal microscope (LSM780, Zeiss, Germany). Di-8 ANEPPS was excited at 488 nm and its emission recorded at 507–690 nm. Image stacks were acquired using a 63 × oil immersion lens (Zeiss Plan Apochromat, Na 1.4) with gradual laser power increase to correct for depth-dependent signal attenuation. The transverse-axial tubular system (TATS) was identified and quantified by custom software scripts, using Matlab (Mathworks, versions 2022a or higher) and the Insight Toolkit (version 4.8) as described earlier [40]. As a measure of TATS density, we calculated the volume fraction of t-tubules inside the cell as well as the mean distance of each intracellular voxel to its closest t-tubule. The Fourier transform was used to calculate the spectral density as a measure of TATS regularity.

### Assessment of mitochondrial function

Isolated mouse cardiomyocytes were incubated for 1 h with 50 nM tetramethylrhodamin-methylester (TMRM, T668, Thermo Fisher) and 400 nM of MitoTracker Green FM (MTG, M7514, Thermo Fisher). Cells were then imaged with an LSM780 confocal microscope (Zeiss) using a 63 × oil immersion lens (Zeiss Plan Apochromat, NA 1.4) to obtain z-stacks of individual cells. Each stack had a dimension of 1280 × 384 x 50 voxels and yielded a volume of 128 × 38.4 x10 µm^3^ per cell. TMRM and MTG were excited at 561 nm and 480 nm and emission recorded at 569–664 nm and 490–542 nm, respectively. Cells were selected via brightfield microscopy to avoid bias. To confirm the response of TMRM to changes in mitochondrial inner membrane potential, a second batch of cells of each sample and group was incubated with 10 µM of the oxidative phosphorylation uncoupler carbonyl cyanide-p-trifluoromethoxyphenylhydrazone (FCCP, sc-203578, Santa Cruz) and imaging repeated, using the same microscope settings. Laser power, gain, and imaging speed were kept constant during all experiments. From the acquired images, three-dimensional cell masks were created based on cytosolic autofluorescence. Within each cell, the sum of the TMRM and MTG signals was calculated and then divided by each other. The TMRM/MTG signal ratio was used as an indicator of mitochondrial function as done in previous studies [4, 8, 15]. Mitochondrial density was assessed by applying histogram-based thresholds (mode + two standard deviations) to the image within the cell mask and then dividing the number of voxels above the threshold by the total number of voxels in the cell.

### Patch clamping

Transmembrane currents and action potentials were measured in the whole-cell configuration after successful seal formation and rupture. Measurements took place at room temperature in a chamber mounted on the stage of an inverted microscope (Axiovert 25, Zeiss). Patch pipettes were pulled from borosilicate glass (outer diameter 1.5 mm, inner diameter 0.87 mm, Hilgenberg, Germany) using a P-97 Puller (Sutter Instruments, USA). Pipette resistance was 1.5–5 MΩ. Recordings were performed with an EPC 10 amplifier (HEKA Elektronik, Germany), driven and operated by PULSE software (HEKA Elektronik, Germany). Action potentials were recorded in *Patch clamp solution* right after rupturing in current-clamp mode at a pacing rate of 1 Hz. The 30th AP was used for analysis. The cells’ resting potential was not manipulated by a holding current. Patch pipettes were filled with *Pipette solution*. For measuring transmembrane currents, the amplifier was switched to voltage clamp mode and cells were superfused with inhibitors (Cd^2+^ or Ba^2+^). Serial resistance was compensated by 85% and kept below 10 MΩ by gentle application of air pressure to a tube connected to the pipette.

To investigate I_to_, cardiomyocytes were clamped from a holding potential of −90 mV to test potentials between + 60 mV and −80 mV in steps of −20 mV and for 600 ms. Interfering Ca^2+^ currents were blocked with 300 µM Cd^2+^. A pre-pulse to inactivate the fast Na^+^ current was added. I_to_ was assessed as the difference between current peak and the remaining current at the end of the pulse. To determine the steady-state activation of I_to_, we normalized conductivity in each test potential to the conductivity at V_Pip_ =  + 60 mV. The normalized conductivities were fit to the Boltzmann equation (see supplemental material). I_to_ steady-state inactivation was assessed with a two-pulse protocol: From a holding potential of −90 mV, a 600 ms conditioning pulse from V_Pip_ = −80 mV to 10 mV was followed by a test pulse to V_Pip_ =  + 60 mV. I_to_ amplitude of the test pulse was normalized to I_to_ recorded after the conditioning pulse to V_Pip_ = −80 mV. Steady-state inactivation was fit using the Boltzmann equation (see supplemental material). To assess recovery from inactivation, another two-pulse protocol was used: A first pulse from a holding potential of −90 mV to a V_Pip_ =  + 60 mV was followed by an interval of repolarization to −90 mV and a second pulse to + 60 mV. The repolarization interval was increased stepwise by a factor of 1.5. I_to_ measured during the second pulse was normalized to I_to_ measured during the first pulse. The normalized currents were fit to a bi-phase association (see supplemental materials). To assess I_K_, the mean at the end of the I_to_ pulses was used. To assess I_K1_, the Ba^2+^-sensitive current was measured. From a holding potential of −90 mV, cells were clamped to test potentials between −120 mV and + 40 mV for 600 ms. The protocol was then repeated in the presence of 1 mmol/l Ba^2+^ in the *Patch clamp solution*. The difference of the currents at the end of the respective 600 ms pulses was identified as I_K1_.

### Ca^2+^ imaging and contraction analysis

Isolated cells were incubated in *Ca*^*2*+^ *imaging solution*, supplemented with 1 µmol/L Fura-2-AM and 0.1% pluronic acid at room temperature for 20 min. Afterwards, cells were washed and resuspended in 1 ml *Ca*^*2*+^ *imaging solution*. Measurements took place in a chamber on top of an inverted microscope (Leica, Germany) with continuous superfusion (160–250 ml/h) at 34–35 °C. Field stimulation was constantly applied with a voltage generator (MyoPacer, IonOptix, USA) and cells were alternatively excited at 340 and 380 nm with a switching rate of 200 Hz (dual excitation, Hyper-switch, IonOptix, USA). The fluorescence intensity ratio 340/380 served as an indicator for intracellular Ca^2+^. To reach steady-state conditions, cells were paced continuously for at least 1 min before recordings started. Simultaneously, the sarcomere length was quantified based on real-time analysis of the frequency spectrum in the transmitted light image by IonWizard 6.6 software package (IonOptix, Westwood, MA, USA). Electrical stimuli and temperature were recorded. Cells were stimulated with increasing frequencies of 0.5, 1, 1.5, 2, 2.5, 3, and 4 Hz. Each interval contained at least 24 stimulations or at least 12 s.

The resulting time-series data were analyzed with custom-written Matlab scripts (Mathworks, USA, version 2022a and higher). First, peak detection of the voltage signal was used to determine stimulation times. The raw signals (F340/380 ratio and sarcomere length) were then filtered with a moving median filter (radius 10) and a moving mean filter (radius 5). The minimum value of each time-series (offset) was subtracted and the noise amplitude A_noise_ detected by searching the maximum within the interval 200 ms preceding a stimulus at pacing frequencies ≤ 1 Hz. Next, to identify individual Ca^2+^ signals or contractions, peak detection was applied, using a minimum peak height of 2•A_noise_. If no peak was detected, the stimulus was identified as not captured. Otherwise, the signal was further analyzed to quantify its diastolic level, amplitude, time to peak, time to baseline, and Ca^2+^ transient duration.

Parameters from six successive stimulations at the end of an interval of a certain stimulation frequency were averaged and used as representative value for one cell at the respective frequency.

### RNA sequencing

Following isolation, cellular viability was checked by light microscopy. Cardiomyocyte preparations with at least 50% clearly elongated, rod-shaped cross-striated cells were used for RNA sequencing. The cell suspensions were centrifuged, the supernatant was discarded, and the cell pellet was frozen at − 80° C. Total RNA was isolated with a commercial kit (Nucleospin, Machery Nagel, Germany) according to the manufacturer’s instructions. After quality check, the resulting mRNA was processed into a library with the Illumina Stranded mRNA Kit (Illumina, San Diego, USA). The library was sequenced as 150 + 150 bp paired-end reads on an Illumina Novaseq 6000 platform. Each sample yielded 45–54 Mio raw reads. After removal of optical and proximal duplicates and adapter and quality trimming, the resulting reads were aligned against the GRCm38 reference genome with the STAR aligner software (version 2.7.10 a) [11]. Transcript quantification was performed with Salmon 1.9 software.

#### Pathway and gene set enrichment analysis

Statistical analysis of normalized pseudocounts was performed with the DEseq2 package [28] Version 1.30 in the R statistics environment (Version 4.0.3) library. Data were further analyzed and visualized with the use of QIAGEN IPA (QIAGEN Inc.). Additionally, the PROGENy package (v1.26) was applied to analyze pathway activity [38], using the top 1000 genes of the mouse model of each pathway. Log-transformed normalized counts and the Wald statistic were used for analyzing activity in single samples and in grouped comparisons, respectively. Prior to the analysis, invalid (nan) or duplicate entries were removed. Heatmaps and scatterplots were created using the pheatmap (v1.0.12) and ggplot2 (v3.5.1) packages, respectively.

### Statistics

If not otherwise indicated, data are presented as mean ± SEM. Welch’s t test was used to test for differences in group means. Multiple comparison correction was applied for each shown parameter if more than two groups were compared with each other, using the Holm–Bonferroni method.

To test for equivalence, the two one-sided t tests (TOST) [19, 25, 39] were applied. In short, an upper mean difference (Δ_U_) and a lower mean difference (Δ_L_) are defined as thresholds for relevance and compared against the statistically inferred confidence bounds of the effect size (Δ). In the case of -Δ_L_ < Δ < Δ_U_, the mean of the two investigated groups is considered to be equivalent, i.e., the hypothesis that an effect large enough to be considered relevant is rejected. The level of significance (alpha) was set to 0.05. Effect sizes of ± 30% were considered relevant.

## Results

### Cell damage in cold storage

To assess cardiomyocyte damage, troponin T levels were measured in the cold storage solution after 24 h (*n* = 3) and, in two cases, also after 48 h. As a positive control, whole mouse hearts were homogenized to determine the total troponin T content, presuming that homogenization releases all troponin T. In none of the samples'solution did troponin T reach the detection limit (0.045 μg/L). Given the storage solution volume (40 ml), the maximum amount of released troponin was ≤ 0.0018 μg. The troponin T content in homogenized hearts (presumed total amount) was 0.35 ± 0.05 μg per heart, indicating that cardiomyocyte damage was less than 0.5% during the first 24 h of preservation (Supplemental Table 1).

### Number of isolated cells

We were consistently able to isolate adequate amounts of left-ventricular myocytes from all three groups. As expected, immediate isolation yielded large numbers of viable, cross-striated cells as observed and quantified earlier [13]. This was also true for the CSP and CS groups (after 24 h), while a significant reduction in the yield occurred after 48 h and 72 h (CSP48h and CSP72h, see Supplemental Fig. 1). In total, we carried out cardiomyocyte isolations from 31 CTRL hearts (14 male, 17 female), 25 CSP hearts (12 male, 13 female) and 27 CS hearts (13 male, 14 female), 2 CSP48h hearts and 2 CSP72h hearts (1 male, 1 female each). Mean animal age was 107.6 ± 6 days in CTRL, 120 ± 5 days in CSP, 100.2 ± 5 days in CS, 89 ± 1 days in CSP48h and 70 ± 2 for CSP72h. In some isolations, the fraction of cross-striated rod-shaped myocytes was visibly lower than 50%. This was the case for 4 CTRL, 10 CSP, 4 CS hearts, and all CSP48h and CSP72h hearts, but cells from all isolations were used for subsequent experiments.

**Fig. 1:**
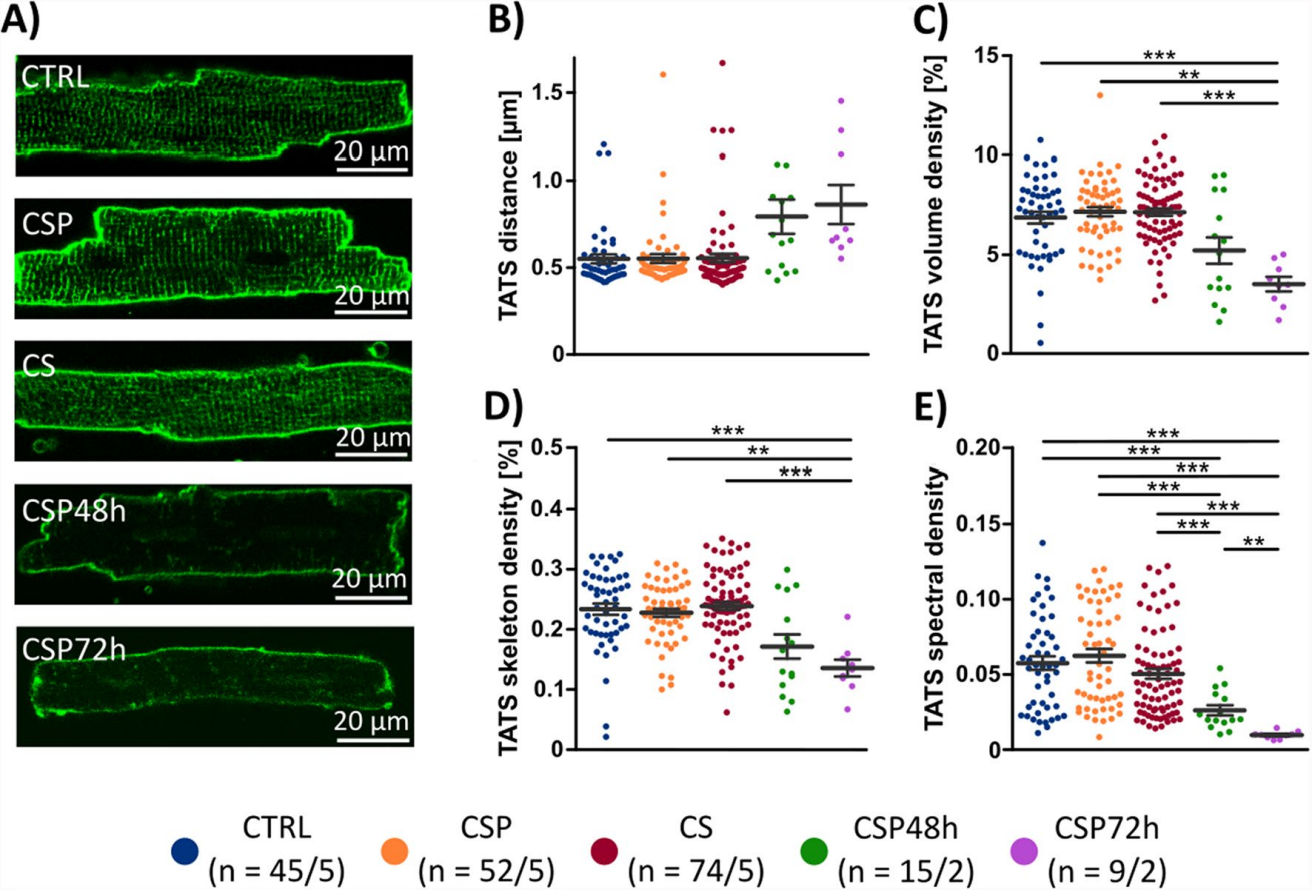
Transverse–axial tubular system. Cardiomyocytes isolated from CTRL (blue, *n* = 45/5 cells/hearts), CSP (orange, *n* = 52/5), CS hearts (red, *n* = 74/5), CSP48h hearts (green, *n* = 15/2), and CSP72h hearts (purple, *n* = 9/2) were stained with the membrane dye Di-8-ANEPPS and imaged with confocal microscopy to assess the transverse–axial tubular system (TATS) **A** Example images of the five groups. **B** Mean intracellular distance of transverse-axial tubules **C** TATS volume density, **D** TATS skeleton density. Densities are provided as the percentage of the cell volume occupied by the TATS or the TATS skeleton, respectively. **E** TATS spectral density, a measure of t-tubule regularity. Statistical test used: unpaired, two-sided Welch’s t test, * p < 0.05, ** *p* < 0.01, *** *p* < 0.001

### Transverse–axial tubular system

The transverse–axial tubular system (TATS) is an important component of cardiomyocyte excitation–contraction coupling. It can also be considered as an indicator of structural integrity, because it was shown to quickly deteriorate in cell culture after cardiomyocyte isolation [1, 26]. Figure 1A shows in representative example cells that the TATS was comparable in the three experimental groups CTRL, CSP, and CS. This was confirmed by quantitative analysis. We calculated the intracellular t-tubule distance (Fig. 1B), volume density (Fig. 1C), skeleton density (Fig. 1D), and TATS spectral density (Fig. 1E). None of these parameters was significantly changed, and the two one-sided t tests (TOST) indicated equivalence of the CTRL, CSP, and CS group (Supplemental Table 2). These results indicate a preservation of the TATS during the 24 h storage period. Extending the storage period leads from a trend after 48 h to significant changes after 72 h in TATS density and regularity (Fig. 1C–E).

### Survival and structural maintenance in cell culture

To determine the effects of cold storage on the survival rate and t-tubule degradation in cell culture, cell counting and t-tubule imaging were repeated for a subset of CTRL and CS cardiomyocytes 6 h and 24 h after cell isolation. In both groups, the cell count decreased with culture time to approx. 60–80% of their initial values after 6 h and 21–38% after 24 h. Although no significant difference was found between CS and CTRL, cell counts appeared slightly higher in CTRL than in CS (Supplemental Fig. 2A) and the TOST did not indicate equivalence (Supplemental Table 2). TATS density, as expected, decreased with culture time, indicated by increased intracellular TATS distances. However, only marginal differences were found between CS and CTRL (Supplemental Fig. 3). TATS volume and skeleton density were slightly lower in CS than in CTRL after 24 h, but not after 6 h. Other parameters, such as spectral density and TATS distance, were not altered at any time point. Fitting to these results, the TOST indicated equivalence (Supplemental Table 2).

**Fig. 2:**
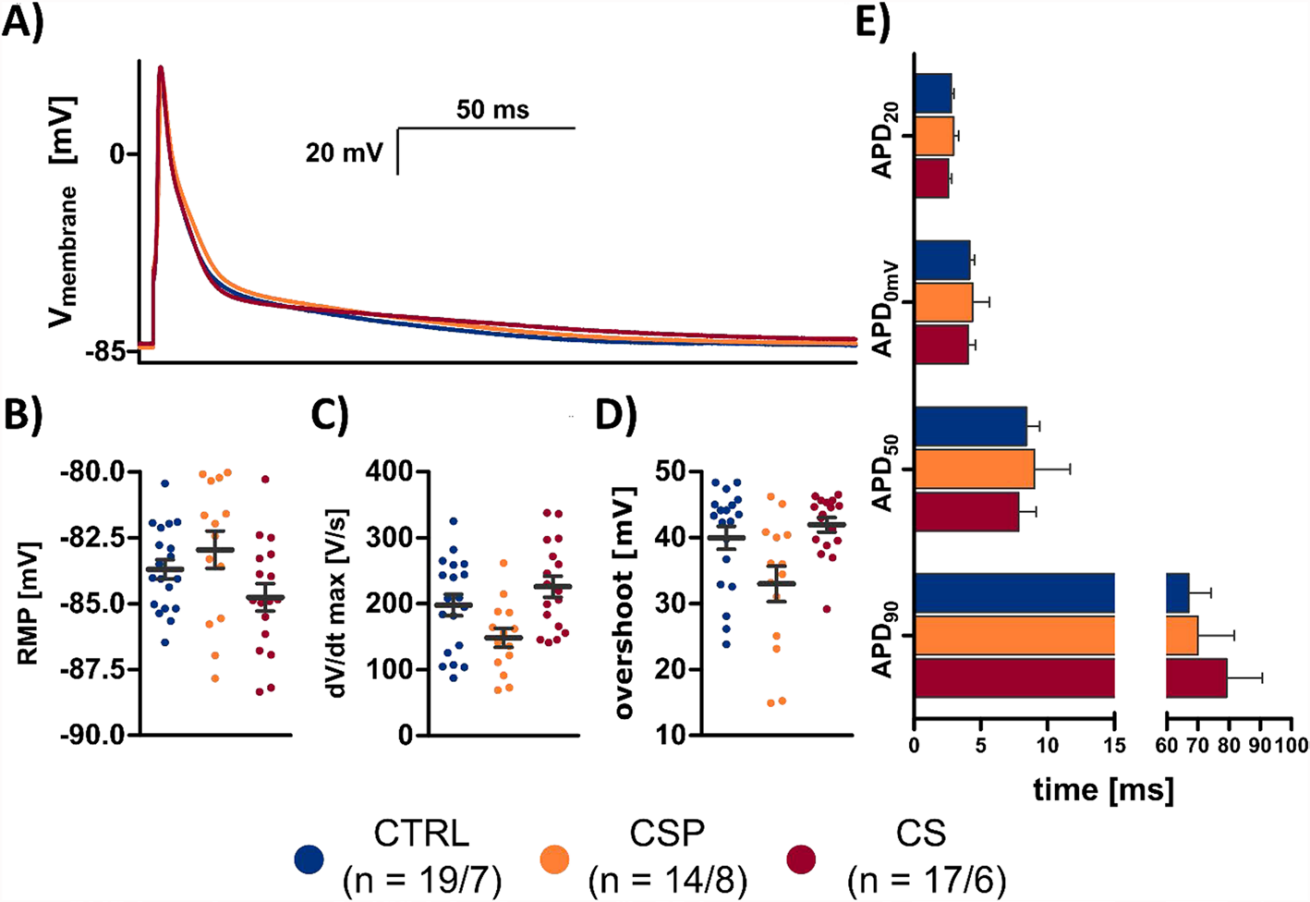
Steady-state action potentials. Recordings in zero-current-clamp mode at 1 Hz pacing in CTRL (*n* = 19/7 cells/hearts, blue), CSP (*n* = 14/8, orange), and CS (*n* = 17/6, red). **A** Representative APs during 1 Hz pacing. The initial stimulation artifact was removed for clarity. **B** Resting membrane potential (RMP). **C** Maximum upstroke velocity (dV/dt_max_). **D** Overshoot during the depolarization phase. **E** Action potential duration (APD) measured at 20%, 50%, and 90% repolarization and repolarization to 0 mV. Statistical test used: unpaired, two-sided Welch’s *t* test, *p *> 0.05 in all comparisons between CTRL; CSP and CS

**Fig. 3:**
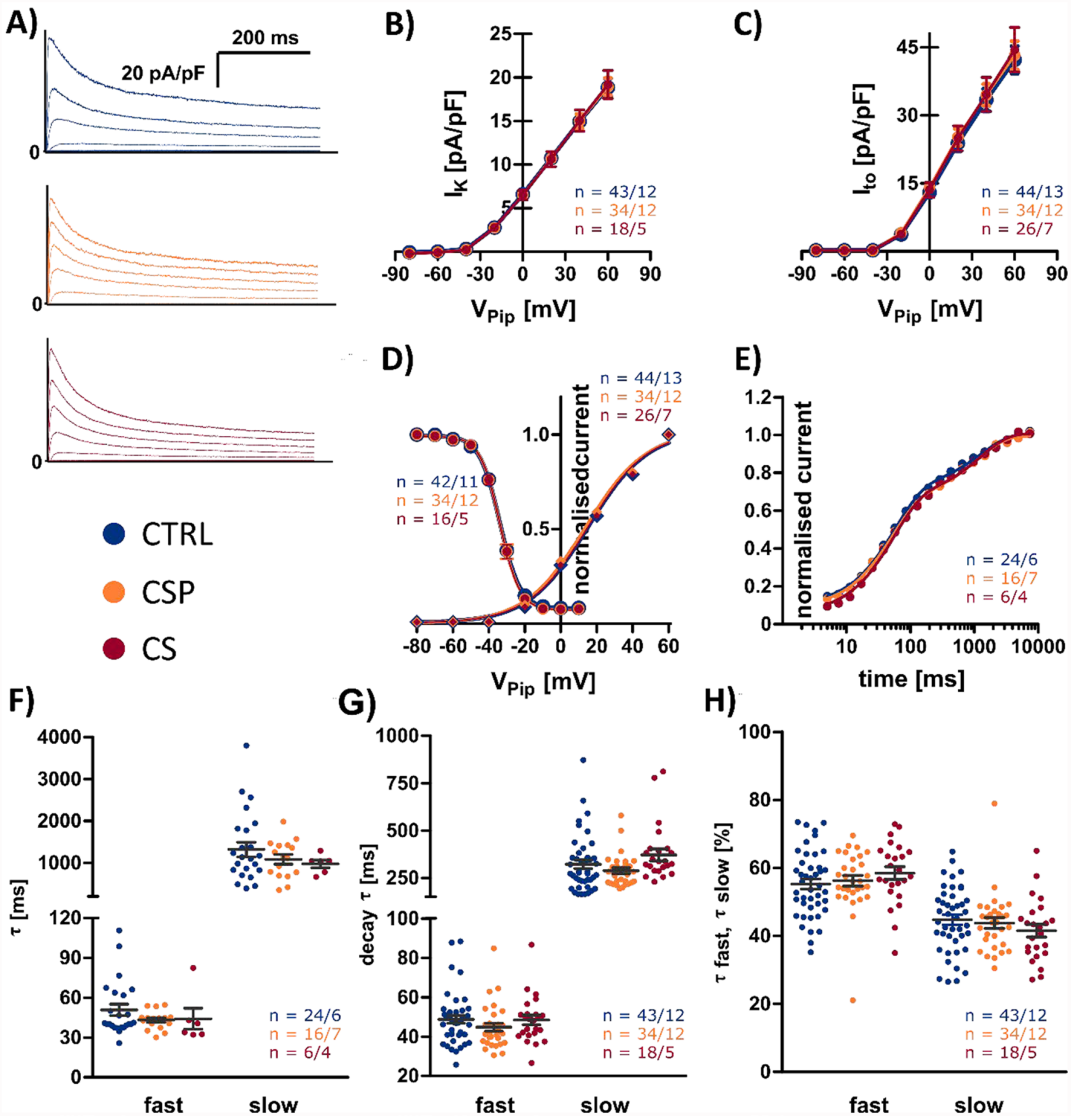
Transient outward (I_to_) and delayed rectifier current (I_K_). **A** Representative traces of whole-cell outward K^+^ currents, normalized to cell capacitance of CTRL (*n* = 24–34/6–13 cells/hearts, blue), CSP (*n* = 16–34/7–12, orange) and CS (*n* = 6–26/4–7, red). From a holding potential of V_Pip_ = −90 mV, cells were clamped to test potentials. A pre-pulse to inactivate the Na^+^ current was applied. **B** I_K_ current–voltage relationship obtained from measurements as shown in A. **C** I_to_ current–voltage relationships obtained from measurements as shown in A. **D** Steady-state inactivation of I_to_ fitted by the Boltzmann equation. A 600 ms conditioning pulse from V_Pip_ = − 80 mV to + 10 mV was followed by a test pulse to V_Pip_ =  + 60 mV. The resulting I_to_ amplitude was normalized to the respective amplitude at V_Pip_ = −80 mV. **E)** I_to_ recovery from inactivation, determined by a two-pulse protocol, separated by repolarization to V_Pip_ = −90 mV. I_to_ of the second pulse was normalized to the I_to_ of the first pulse and plotted against the inter-pulse interval. **F** I_to_ recovery time constants obtained from bi-exponential fitting as explained in E. **G** I_to_ inactivation time constants “τ fast” and “τ slow” obtained bi-exponential fitting of I_to_ currents at V_Pip_ = 60 mV. **H** Contribution of the fast (τ_1_) and slow component (τ_2_) to I_to_ inactivation. Statistical test used: unpaired, two-sided Welch’s t test, p > 0.05 in all comparisons between CTRL, CSP, and CS

### Electrophysiology

#### Action potentials

Action potentials (AP) from isolated cardiomyocytes were recorded in whole-cell current-clamp mode at room temperature. To reach steady-state conditions, we paced for 30 s at 1 Hz. Figure 2A shows representative action potential recordings obtained from the three groups. The resting membrane potentials (RMPs) varied from − 80 to − 88 mV, the typical range of ventricular myocytes, and were not significantly different in the storage groups CSP and CS when compared with CTRL (Fig. 2B). Maximum upstroke velocity (Fig. 2C) during the initial depolarization phase appeared reduced in the CSP group (148.44 ± 13.97 mV/ms) in comparison to CTRL and CS (198.1 ± 15.7 and 225.9 ± 15.6 mV/ms respectively), although this trend did not reach statistical significance. The TOST indicated that the CSP and CS groups were not equivalent with CTRL for this parameter (Supplemental Table 2). A similar result was obtained when analyzing the AP overshoot during the depolarization phase (Fig. 2D). The overshoot appeared less pronounced in CSP when compared with CTRL or CS. Because both maximum upstroke velocity and overshoot depend on Na^+^ channel availability, which can be altered by small changes in the RMP, we investigated the correlation between RMP and these two parameters. We found a significant and steep negative correlation between upstroke velocity and RMP (*r* = − 0.59, *p* < 0.01) as well as overshoot and RMP (*r* = − 0.5, *p* < 0.01, Supplemental Figs. 4 and 5), suggesting that small RMP differences resulted in relatively large differences of maximum upstroke velocity and overshoot. AP duration (APD) was in the typical range of adult rat cardiomyocytes at room temperature and almost identical in all groups (Fig. 2E). We found no significant differences in APD_20_, APD_50_, APD_90_, or APD_0mV_.

**Fig. 4:**
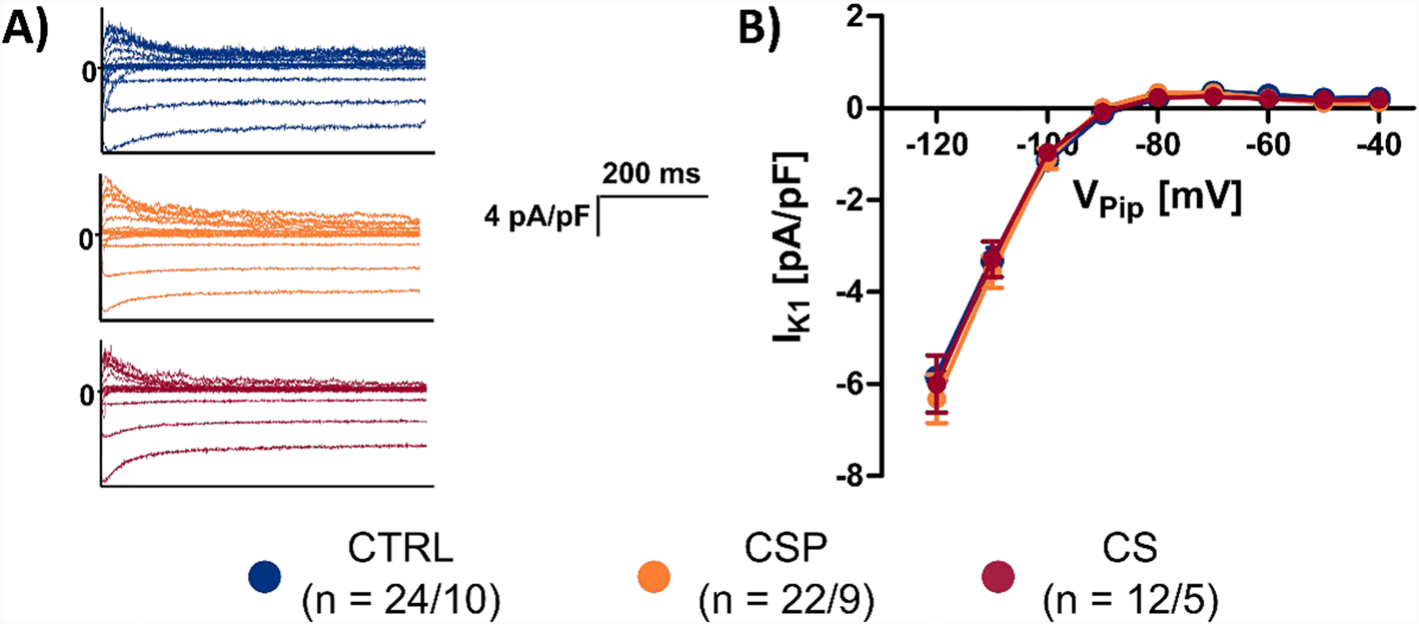
Inwardly rectifying K^+^ currents (I_K1_). **A** Traces show examples of the Ba^2+^-sensitive current, obtained from the difference of current recordings before and after application of Ba^2+^ in a CTRL (*n* = 24 cells/10 hearts, blue), CSP (*n* = 22/9, orange), and CS (*n* = 12/5, red) cardiomyocyte. From a holding potential of − 90 mV cells were clamped to potentials between − 120 and − 40 mV in 10 mV steps for 600 ms. **B** Mean current–voltage relationships of I_K1_ from measurements shown in A. I_K1_ was identified as the Ba^2+^-sensitive current at the end of the 600 ms pulse. Statistical test used: unpaired, two-sided Welch’s *t* test, * *p* > 0.05 in all comparisons between CTRL, CSP, and CS

**Fig. 5:**
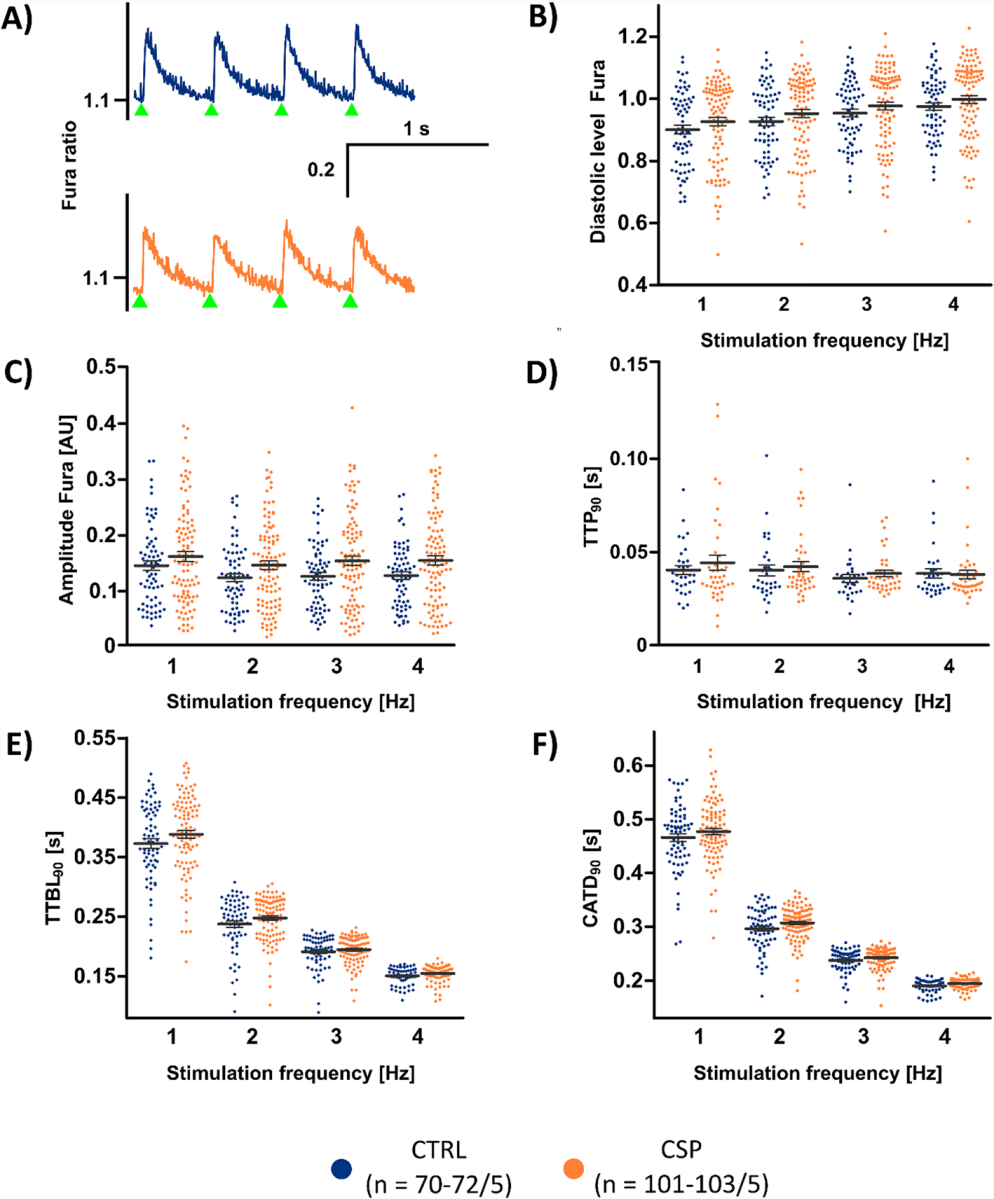
Intracellular Ca^2+^ recordings. **A** Representative recordings of the Fura-2 signal (emission ratio at excitation wavelengths 340 nm and 380 nm) at 2 Hz pacing of CTRL (blue, n = 70–72/5 cells/hearts) and CSP cells (orange, *n* = 101–103/5). **B** Diastolic Fura signal at given pacing frequencies. **C** Amplitude of Fura signal upon electrical field stimulation at given frequencies. **D** Time to peak (TTP_90_), measured from 10% to the maximum of the signal during the upstroke phase of the Ca^2+^ transient. **E** Time to baseline (TTBL_90_), measured from the maximum to 10% of the signal during the decay phase of the Ca^2+^ transient. **F** Ca^2+^ transient duration, measured from 10% of the signal during the upstroke phase until 10% of the signal during the decay phase (CATD_90_ = TTP_90_ + TTBL_90_). Statistical test used: unpaired, two-sided Welch’s *t* test, *p* > 0.05 in all comparisons between CTRL and CSP

#### *K*^+^*currents*

##### *I*_*K*_

I_K_ was identified as the current at the end of recordings used for I_to_ (Fig. 3A). At a test potential of 60 mV, I_K_ was 18.9 ± 1.1 pA/pF in CTRL (n = 43/13 cells/hearts), 18.9 ± 1.0 pA/pF in CSP (n = 34/12), and 19.2 ± 1.6 pA/pF in CS (*n* = 26/7). Thus, I_k_ was nearly identical in all groups and we could not detect any significant differences (Fig. 3B). The TOST indicated equivalence (Supplemental Table 2).

##### *I*_*to*_

We investigated the transient outward potassium current (I_to_) in all three groups in whole-cell voltage clamp mode by blocking the L-type Ca^2+^ current with CdCl_2_ and applying test pulses from a holding potential to + 60 mV and − 80 mV in steps of −20 mV from a holding potential of − 90 mV. I_to_ was quantified as the difference between the peak current and the current at the end of the pulse (Fig. 3A). Compared to CTRL, we found no significant differences in the CSP and CS groups (Fig. 3C). I_to_ current density at V_Pip_ =  + 60 mV was 42.3 ± 3.0 pA/pF in CTRL (*n* = 44/13 cells/hearts), 43.2 ± 3.2 pA/pF in CSP (*n* = 34/12) and 44.5 ± 4.9 pA/pF in CS (*n* = 26/7). In line with this result, the TOST indicated equivalence (Supplemental Table 2). The current traces at V_Pip_ =  + 60 mV showed a bi-exponential decay with a fast and a slow component. Curve fitting yielded no significant differences in the time constants (τ_fast_ = 48.7 ± 2.0 ms in CTRL, 44.8 ± 2.1 ms in CSP, 48.5 ± 2.4 in CS; τ_slow_ = 322.0 ± 22.6 ms in CTRL, 287.9 ± 15.6 in CSP, 371.4 ± 31.6 in CS, Fig. 3G). Similarly, the contribution of both components (Fig. 3H) was not significantly different (c_1_ = 55.2 ± 4% in CTRL, 56.2 ± 5% in CSP, 58.5 ± 5% in CS). The normalized current densities measured during the inactivation protocol were fit to the Boltzmann equation (Fig. 3D). The half-inactivation potential was similar in all three groups with −34.0 ± 0.01 mV in CTRL, − 33.9 ± 0.4 mV in CSP and − 34.0 ± 0.4 mV in CS. The TOST indicated equivalence (Supplemental Table 2). Time-dependent recovery from inactivation revealed a K_fast_ of 50.5 ± 4.2 ms in CTRL, 43.1 ± 1.8 ms in CSP, and 44.1 ± 7.9 ms in CS (Fig. 3F). K_slow_ was 1319.0 ± 175.0 ms in CTRL, 1086.0 ± 112.5 ms in CSP and 978.8 ± 91.3 ms in CS. There was no significant difference in the two constants, compared to the CTRL group.

##### *I*_*K1*_

From a holding potential of − 90 mV, cells were clamped to test potentials between − 120 mV and − 40 mV in steps of 10 mV for 600 ms. The pulse protocol was repeated after superfusion with 1 mM BaCl_2_ to calculate I_K1_ as the current difference before and after Ba^2+^ superfusion at the end of the voltage step (Fig. 4A). The resulting current–voltage relationships are shown in Fig. 4B. At − 120 mV, current density reached −5.9 ± 0.5 pA/pF in CTRL (*n* = 24/10 cells/hearts), − 6.3 ± 0.5 pA/pF in CSP (*n* = 22/9) and − 6.0 ± 0.6 pA/pF in CS (*n* = 12/5). No significant differences were found at any of the tested voltages between the three groups. The TOST indicated equivalence (see Supplemental Table 2).

### Membrane capacitance

During current patch clamp recordings, the cell capacitance, which is directly related to the membrane surface area, was measured. The surface area depends on cell size and TATS density. Because the TATS was shown to be unaltered in CSP an CS when compared with CTRL (Fig. 1), cell capacitance was considered as an indicator of cell size differences. Cell capacitance was 172.6 ± 9.7 pF in CTRL (*n* = 41 cells/16 animals), 167.9 ± 10.2 pF in CSP (*n* = 40/15) and 149.9 ± 9.6 pF in CS (*n* = 26/6). A significant difference to the CTRL group was not detected, but TOST did not indicate equivalence (Supplemental Table 2).

### Intracellular Ca^2+^ signal

To assess excitation–contraction coupling, isolated cardiomyocytes of the CTRL and CSP groups were loaded with the ratiometric Ca^2+^ indicator Fura-2 and then stimulated on the stage of a microscope at 34–35 ˚C. Examples of Ca^2+^ transients at 2 Hz pacing rate are shown in Fig. 5A, suggesting no major differences in the Ca^2+^ signal between CTRL and CSP cells. This was confirmed by quantitative analysis of the diastolic level (Fig. 5B) and Fura signal amplitude (Fig. 5C). The diastolic level increased with pacing frequency, while the amplitude remained nearly constant. There were no significant differences between the two groups over the whole range of pacing frequencies (1–4 Hz). Also, the kinetic parameters of the Ca^2+^ transients were very similar both groups (Fig. 5D–F). Time to peak (TTP), time to baseline (TTBL), and Ca^2+^ transient duration (CATD) were not significantly different in CSP cells and CTRL cells. Fitting to this, the TOST indicated equivalence of the parameters (Supplemental Table 3) at the majority of tested pacing frequencies. While TTP remained relatively stable, TTBL decreased with increasing pacing rate, resulting in an approximately 40% reduction at 4 Hz when compared with 1 Hz in both groups. As a result, CATD was lower at 4 Hz than at 1 Hz. When comparing CS with CTRL, we found significantly higher diastolic Fura signals and shorter TTP in CS cells. Fura amplitudes and CATD, however, were not different (Supplemental Fig. 6). Overall, these results suggest that the Ca^2+^ signal amplitude, kinetics, and response to pacing rate are preserved for 24 h under our experimental conditions. Experiments with CSP48h resulted in detectable amplitudes in only a single out of 14 cells and in CSP72h in only 2 out of 19 cells (two isolations each, data not shown).

**Fig. 6:**
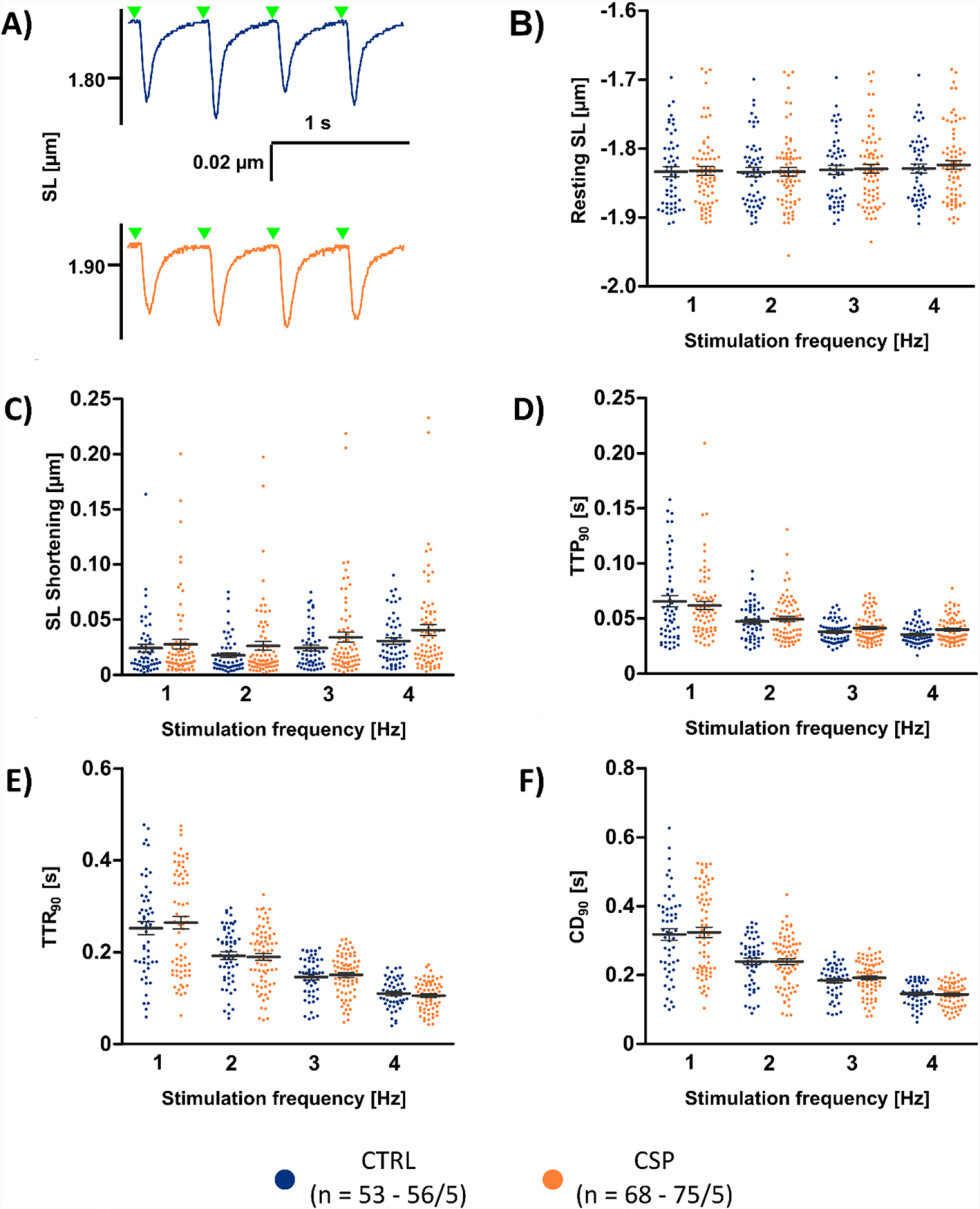
Contraction parameters. **A** Representative examples of sarcomere length (SL) changes upon electrical stimulation at 2 Hz in the CTRL (blue, *n* = 53–56/5 cells/hearts) and CSP group (orange, *n* = 68–75/5), determined by Fourier analysis of striations visible in brightfield microscopy. Time of stimulation is indicated by green triangles. **B** Diastolic SL at different pacing frequencies. **C** Maximum SL shortening (amplitude) at different pacing frequencies. **D** Time to peak (TTP_90_), measured from 10% to the maximum of the contraction. **E** Time to relaxation (TTR_90_), measured from the point of maximum shortening to 10% of the diastolic SL during relaxation. **F** Contraction duration (CD_90_), measured from 10% of the shortening during the contraction phase until 10% of the shortening during the relaxation phase (CD_90_ = TTP_90_ + TTR_90_). Statistical test used: unpaired, two-sided Welch’s t test, *p* > 0.05 in all comparisons between CTRL and CSP

### Cardiomyocyte contraction

Sarcomere length was simultaneously recorded with the Ca^2+^ signal, determined by Fourier analysis of the cross striations visible in brightfield microscopy. Examples of the sarcomere length changes at 2 Hz pacing rate are shown in Fig. 6A. Diastolic sarcomere length in the CSP group was equivalent to CTRL at all tested pacing frequencies (Fig. 6B, Supplemental Table 4). Sarcomere length shortening (Fig. 6C) was also not significantly different, although the TOST did not indicate significance (Supplemental Table 4)—most likely a result from some outliers. In both groups, the maximum shortening increased with pacing rate, indicating a preserved positive force-frequency relationship after cold storage. Parameters of contraction kinetics (TTP, TTR, and CD) were unaltered and equivalent at nearly all pacing frequencies (Fig. 6D-F, Supplemental Table 4). However, similar to our findings for the Ca^2+^ signals, the CS group showed some differences vs CTRL (Supplemental Fig. 7). The resting sarcomere length was higher and sarcomere shortening showed a trend toward lower values, whereas contraction kinetics were not different in CSP. These results indicate that cold storage, especially with prior perfusion, preserves the contraction and relaxation of cardiac myocytes.

**Fig. 7:**
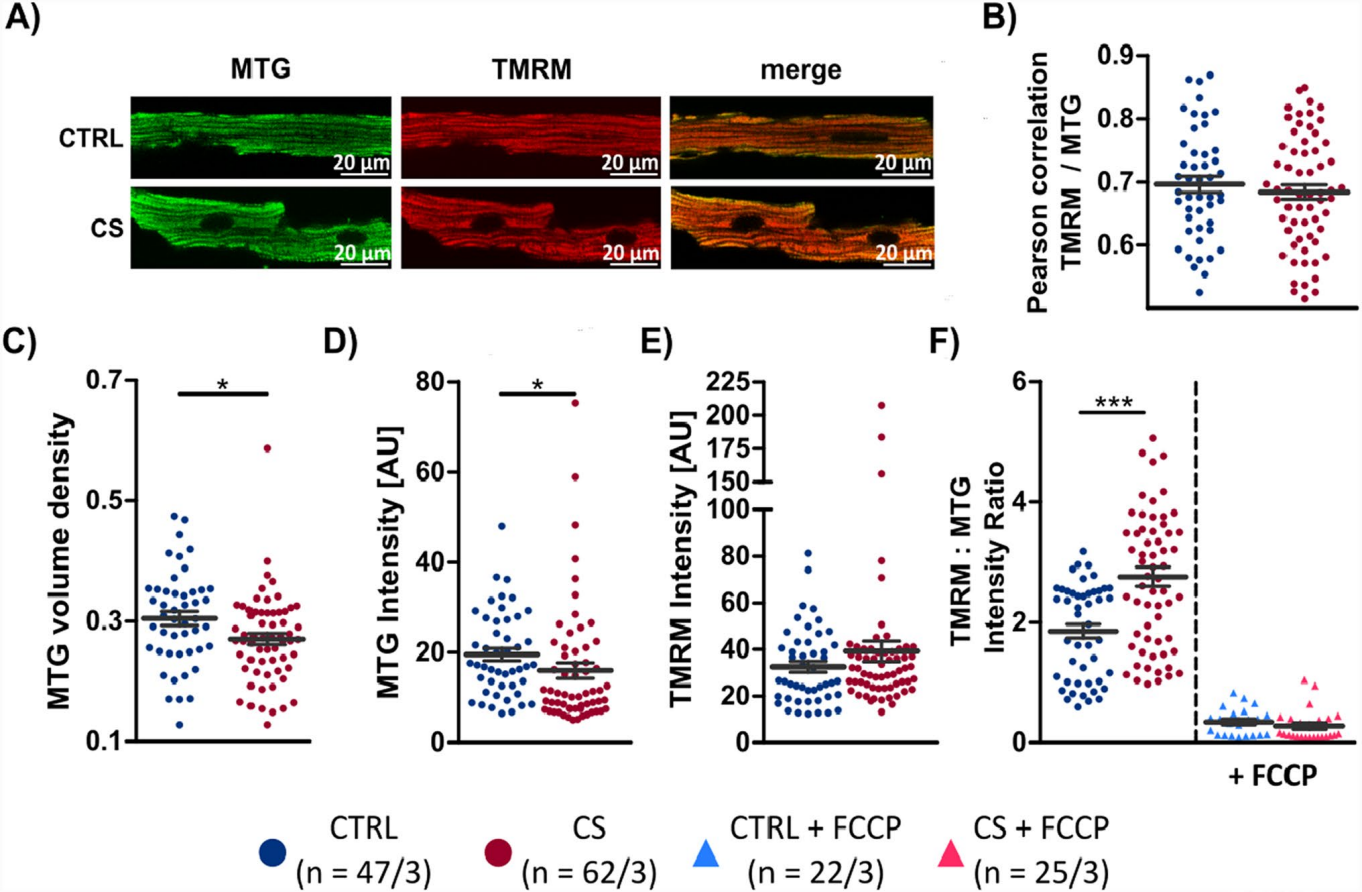
Assessment of mitochondrial function. Cardiomyocytes isolated from CTRL (blue, *n* = 47/3 hearts/cells) and CS (red, *n* = 62/3) hearts were stained with Mitotracker Green (MTG) and tetramethylrhodamin-methylester (TMRM) and imaged with confocal microscopy. **A** Example images of the two groups with MTG (green), TMRM (red), and merged signals (overlap: yellow). **B** Pearson correlation coefficient of the TMRM and MTG signals in each cardiomyocyte. **C** Fraction of the cell volume stained with MTG. **D** Mean MTG signal intensity per cell. **E** Mean TMRM intensity per cell. **F** TMRM/MTG signal ratio per cell und normal conditions and after the addition of 10 µM of the ionophore carbonyl cyanide-p-trifluoromethoxyphenylhydrazone (FCCP). Statistical test used: unpaired, two-sided Welch’s t test, * *p* < 0.05, ** *p* < 0.01, *** *p* < 0.001

### Mitochondrial function

To assess mitochondrial health of cardiomyocytes isolated after 24 h of cold storage, we used TMRM as an indicator of the inner mitochondrial membrane potential and Mitotracker Green (MTG) as a mitochondrial marker widely independent of the membrane potential [8]. Imaged cells displayed successful staining with both dyes (Fig. 7A) and a high colocalization as indicated by a Pearson correlation coefficient of 0.7 in both groups (Fig. 7B). MTG occupied approx. 30% of the cell volume, fitting well to reported volume densities of mitochondria in cardiac myocytes [37]. Mitochondrial density was slightly reduced in the CS group (Fig. 7C), while the TOST still indicated equivalence for the stained volume (Supplemental Table 2). The signal intensity for TMRM was comparable in both groups (Fig. 7E). Importantly, the TMRM:MTG ratio was not reduced in CS when compared with CTRL. In contrast, it was increased. The addition of FCCP confirmed the TMRM:MTG ratio as a measure of mitochondrial membrane potential as it caused a substantial decrease in both groups (Fig. 7F). Interestingly, the difference in the TMRM:MTG ratio between CS and CTRL disappeared after adding FCCP, suggesting that differences in loading efficiency did not underlie the higher mean value in CS.

### RNA sequencing

To investigate the effects of 24 h of cold storage on gene expression, we performed RNA sequencing in isolated cells from the 6 CTRL and 6 CSP hearts. RNA from 3 males and 3 females per group were sequenced and analyzed for differential gene expression. Principal component and cluster analysis did not show clustering by group or by sex but indicated one outlier in the CTRL group. Because this outlier was prepared in a different batch than all the other samples, a batch effect was presumed, and the sample excluded from further analysis (Supplemental Fig. 9). Using an adjusted p value of 0.05 as cutoff, we found a total of 128 differentially expressed genes, of which 109 were significantly downregulated and 19 upregulated in the CSP group (Supplemental Table 5). Quiagen gene set enrichment and pathway analysis pointed toward a downregulation of gene sets and pathways associated predominantly with immune cells and inflammation (Supplemental Figs. 10 and 11). This was supported by PROGENy pathway analysis, which suggested that cold preservation suppressed the NFκB inflammatory program (Supplemental Table 6). However, the analysis did not reveal any alterations in genes or signals considered to be directly important for cardiomyocyte function. Specifically, fitting to our functional and structural results, no genes associated with the transverse–axial tubular system (TATS), transmembrane currents or Ca^2+^ handling and contraction were altered (Table 1).

**Table 1:** Comparison of mRNA level for selected genes between CTRL and CSP

Symbol	Description	Log2-fold change vs CTRL	*P*-adjusted
BIN1	Bridging integrator 1	1.05	0.999
CAV3	Caveolin 3	0.94	0.999
JPH2	Junctophilin 2	1.13	0.999
RYR2	Ryanodine Receptor 2	1.17	0.999
CASQ2	Calsequestrin2	0.93	0.999
ATP2A2	SERCA2	1.09	0.999
PLN	Phospholamban	0.98	0.999
SLC8A1	Sodium/calcium exchanger	1.16	0.999
CACNA1C	Pore forming unit of L-type Ca^2+^ channel	1.2	0.999
KCNJ2	Inwardly rectifying K^+^ channel	1.07	0.999
KCNJ12	Inwardly rectifying K^+^ channel	1.09	0.999
KCNJ8	Inwardly rectifying K^+^ channel	1.11	0.999
KCNJ4	ATP sensitive K^+^ channel	0.78	0.999
KCNE1	Voltage gated K^+^ channel	1.1	0.999
KCNQ1	Voltage gated K^+^ channel	1.07	0.999
SCN5A	Voltage gated sodium channel	1.24	0.999
TTN	Cardiac titin	0.90	0.999
TNNI3	Cardiac troponin I3	0.85	0.999
TNNT2	Cardiac troponin T2	0.80	0.999
ACTC1	Cardiac α-actin	0.97	0.999
ACTN1	α-Actinin 1	1.17	0.999
ACTN22	α-Actinin 2	1.14	0.999
MYBPC3	Cardiac myosin-binding protein	0.97	0.999
MYH6	Cardiac α myosin heavy chain	0.97	0.999

mRNA transcription level for selected genes in the CSP group. A collection of genes relevant for electric and mechanical function of cardiomyocytes is displayed.

### Proof of principle in a cardiac disease model

**Fig. 8:**
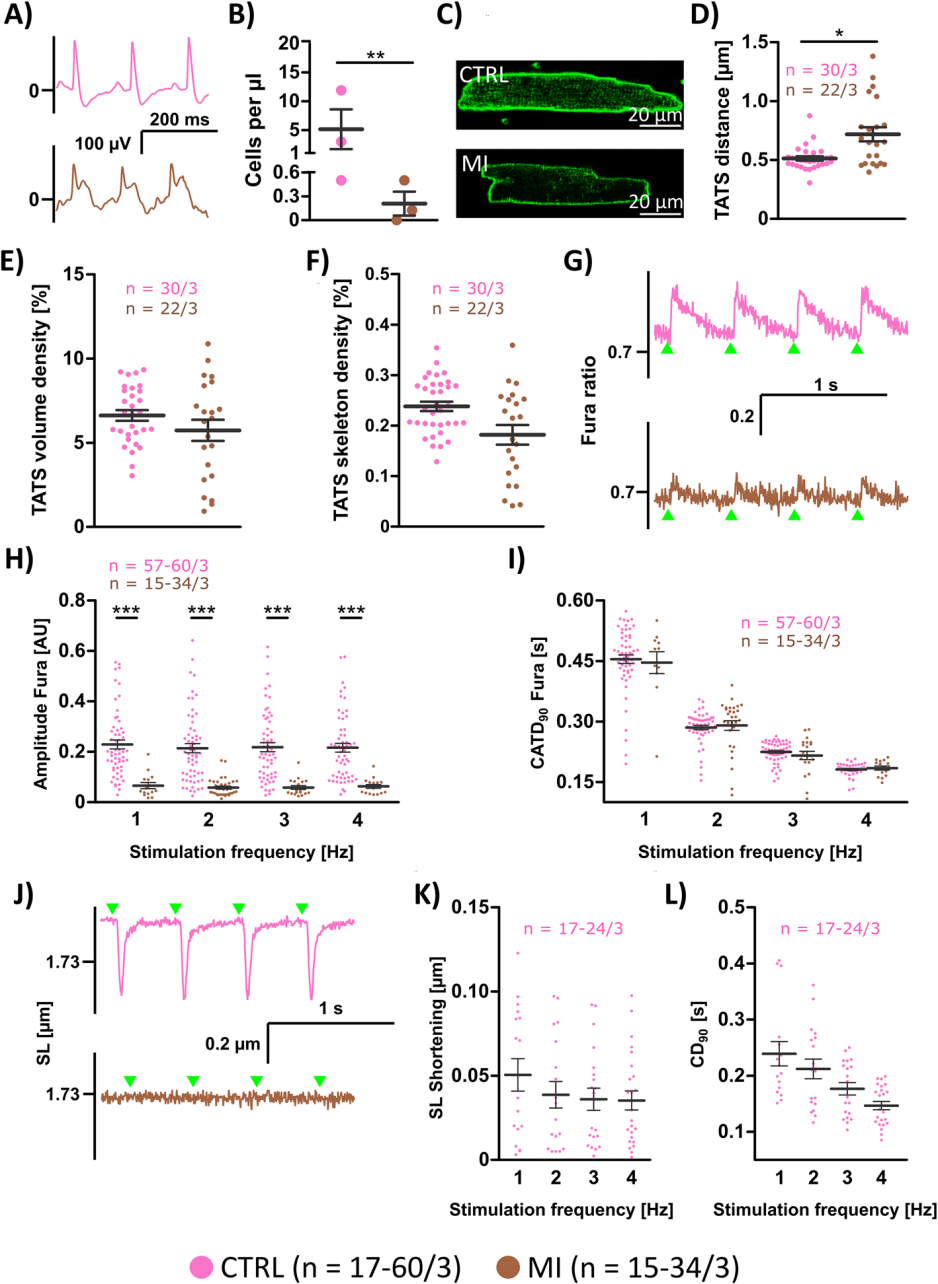
Disease model and proof of principle. Cardiomyocytes from healthy (CTRL) mouse hearts (pink, *n* = 17–60/3 cells/hearts) and infarcted hearts (brown, *n* = 15–34/3 cells/hearts) were isolated and used for subsequent experiments. **A** Exemplary ECG recordings confirmed absence or presence (ST segment elevation) of infarcts. **B** Cell number after three successful isolations from transported hearts. **C** Confocal images of cardiomyocytes stained with Di-8-ANEPPS. **D** Mean intracellular distance of transverse-axial tubules. **E** TATS volume density, **F** TATS skeleton density, and **G** representative recordings of the Fura-2 signal (emission ratio at excitation wavelengths 340 nm and 380 nm) at 2 Hz pacing. **H** Amplitude of Fura signal upon electrical field stimulation at given frequencies. **H** Ca^2+^ transient duration, measured from 10% of the signal during the upstroke phase until 10% of the signal during the decay phase (CATD_90_ = TTP_90_ + TTBL_90_). **J** Representative traces of sarcomere length (SL) changes upon electrical stimulation at 2 Hz pacing. **K** Maximum sarcomere length shortening at different pacing frequencies. **L** Contraction duration, measured from 10% of the shortening during the contraction phase until 10% of the shortening during the relaxation phase (CD_90_ = TTP_90_ + TTR_90_). Statistical test used: unpaired, two-sided Welch's t-test, * p<0.05, ** p<0.01, *** p<0.001.

To test our method in a “real world” setting, we performed experiments with cardiomyocytes isolated from hearts that were shipped overnight, i.e., after approximately 24 h of transport time (Fig. 8). In this series, we compared cells from a control group (CTRL) with cells from a myocardial infarction (MI) model. Effective induction of MI was confirmed by ST segment elevation in the electrocardiogram (Fig. 8A). Mean animal age was 116.7 ± 35 days in CTRL (*N* = 3, 1 male, 2 females) and 121.3 ± 15 days in MI (*N* = 3, 2 males, 1 female). We successfully isolated cardiomyocytes from three hearts in each group (Fig. 8B, C), while one attempt to isolate cells from a control heart failed because of a damage in the aorta, which prevented the perfusion with isolation enzymes.

MI cells exhibited decreased amounts of t-tubules, which fits to earlier reports in MI models [41]: While the t-tubular distance was significantly larger in MI (Fig. 8D), differences in the other parameters did not reach statistical significance (Fig. 8E, F). Ca^2+^ signals measured via Fura-2 signals showed a greater amplitude in CTRL than in MI (Fig. 8H) while the duration of the transients was comparable between the two groups (Fig. 8I). Time to peak, however, was prolonged in MI (see Supplemental Fig. 12), fitting well to reduced TATS densities and studies describing slowed Ca^2+^ release post-MI [32]. Upon electrical stimulation, cardiomyocytes of the CTRL group responded well with visible and measurable contractions on a regular basis (Fig. 8J–L). However, out of 34 cells isolated from 3 MI hearts only a single one contracted in the MI group (data not shown).

## Discussion

Reducing the suffering, number and costs of laboratory animals used in cardiac research, improving the flexibility in working hours when performing cardiomyocyte isolations as well as increasing the opportunities for collaborations between laboratories at different institutions are highly desirable goals. These goals could be better attained if longer periods between removal of the heart or cardiac sample and cell isolation would be acceptable. So far, however, most researchers have been insisting on immediate cell isolation to avoid deterioration of the cells. By showing that a 24 h period of cold storage, using a low-cost, easy-to-apply technique, preserves nearly all important functional and structural characteristic as well as gene expression, this study provides evidence that immediate cell isolation is not as critical as previously thought. Importantly, we demonstrate in mouse hearts that coronary perfusion prior to cold storage is not always required. Rather, immersing the heart in *Cold storage solution* immediately after excision is sufficient for preservation as it hardly affects the subsequent cell isolation. Importantly, we verified the described approach by shipping hearts from one institution to another via an overnight courier and demonstrate that successful cell isolations and experiments are still possible. We suggest that this gives researchers and staff without experience in experimental cardiac research the opportunity to store the heart and to ship it to another laboratory.

### Cellular yield and viability

We were able to isolate high-quality cardiomyocytes from all experimental groups within 24 h after the heart removal. The yield was comparable between CTRL, CSP, and CS, although we did not anticoagulate the mice. In CSP, we perfused the heart immediately after retrieval. The coronary arteries were visibly flushed and freed from blood. In the CS group, we immersed the heart immediately into the storage solution, where we often observed one or two contractions before complete cardioplegia. This may have removed part of the blood from the larger coronary vessels. An additional reason why cardiomyocyte isolation worked well in CS is that perfusion of the larger coronary vessels may be sufficient for the effectiveness of the enzymes, as they only need to distribute inside the extracellular fluids to reach the extracellular matrix.

However, a substantial decrease in cellular yield was observed at storage periods lasting 48 h or more. This quantitative loss was accompanied by functional deterioration. Interestingly, this was not detectable by troponin T release into the storage solution, indicating that the damage occurred during the processes of rewarming and cell isolation. Therefore, we suggest to not exceed a storage time of 24 h. Importantly, the observation of structural and functional deterioration verify that our methods were able to detect signs of cell damage.

Cardiomyocytes isolated from CTRL and CS hearts could be successfully kept in culture for up to 24 h after the isolation. While after 6 h, more than 60% of the initially isolated cells were viable, after 24 h only 20–30% were remaining. This fits to other studies where adult mouse cardiac myocytes were cultured without the addition of extracellular matrix proteins or electromechanical uncouplers [22]. It is known that the addition of blebbistatin or BDM to the culture medium, as well as coating of the culture dishes with laminin, fibronectin, or Matrigel improves culture time, viability, and function. However, our aim was to compare general effects of heart preservation prior to cell isolation on viability in culture. The numbers of cells remaining after 6 h and 24 h were sufficient to conduct experiments, such as live-cell imaging (Supplemental Figs. 2 and 3). Thus, up to 24 h of cold storage has only minor effects on cardiomyocyte survival, although we cannot rule out greater effects with longer culture times.

### TATS and excitation–contraction coupling

To investigate the structural integrity of the myocytes, we chose the transverse-axial tubular system (TATS) for two reasons. First, the TATS deteriorates quickly in isolated cardiac myocytes [40]. Second, remodeling and loss of the TATS tremendously affect excitation–contraction coupling [10, 42]. Here, confocal microscopy after Di-8-ANEPPS staining unveiled a dense and regular TATS after 24 h of cold storage, undistinguishable from immediately isolated cells (Fig. 1). This indicates that the TATS in intact myocardium remains stable or disappears at a much slower rate than after myocyte isolation, which is well in line with reports from other studies investigating myocardial microstructure after tissue culture [2, 14, 17]. When investigating the effects of cell culture of 6 h and 24 h on the TATS here, we confirmed previous studies showing the quick deterioration of t-tubules in isolated cardiomyocytes [40] and also verified that our method is able to detect expected TATS changes. Cold storage of the heart prior to cell isolation, however, had only minor effects on the TATS deterioration rate in culture (Supplemental Fig. 3).

Our finding that the TATS was not affected by 24 h of heart storage aligns with comparable Ca^2+^ signal amplitudes in CTRL, CSP, and CS (Fig. 5, Supplemental Fig. 6). While the CSP group was equivalent to CTRL in nearly all parameters, TTP and TTR at low pacing rates were smaller and greater in CS, respectively, when compared with CTRL. However, the differences disappeared at the highest pacing rate (4 Hz), which is closer to the physiological resting heart rate of the mouse. Total Ca^2+^ transient duration was equivalent at all pacing rates tested, indicating preserved Ca^2+^ release and subsequent removal from the cytosol. Accordingly, the magnitude and speed of contraction and relaxation (Fig. 6) did not differ significantly between CTRL and CSP or CS, except at low pacing rates, where sarcomere shortening appeared less pronounced in CS. Of note, the positive frequency response, similar to the force–frequency response, remained positive after the 24 h storage period, while some studies report blunting or reversal of the FFR after 24 h of tissue culture [18]. In summary, we suggest that studying the cardiac TATS and excitation–contraction coupling can be carried out after 24 h of cold heart storage without relevant restrictions when prior perfusion is applied (CSP). However, highly significant differences in TATS features appear between the CSP48h and CSP72h group compared to CTRL, indicating a change in cellular quality, in case of storage periods longer than 24 h. Measurements of Ca^2+^ transients and contractions were no longer possible after 48 h of storage. This finding further underlines our conclusion of relevant changes if the storage period exceeds 24 h.

### Electrophysiology

The AP shape and duration were unchanged after cold storage (Fig. 2). We did not record the fast inward Na^+^ current, but indirectly assessed its function from maximum upstroke velocity and overshoot. Close inspection of Fig. 2C–D suggests a slower maximum upstroke velocity and decreased overshoot in CSP, which could be explained by reduced Na^+^ channel density or, alternatively, reduced Na^+^ channel availability. However, because we did not clamp the resting membrane potential (no current injection), the small changes in resting membrane potential may have reduced Na^+^ channel availability by slightly increasing the fraction of inactivated channels [3]. In fact, we found a significant and steep correlation between RMP and maximum upstroke velocity of the AP with a similar slope in all groups (Supplemental Fig. 4), which supports this explanation and suggests that I_Na_ was similar in the three groups.

We found no significant differences between the storage groups and CTRL for APD_20_, APD_0mV_, APD_50_, and APD_90_. However, the TOST also did not indicate equivalence. This can be explained by a relatively high variance resulting from myocyte–myocyte differences and noise from the method of measurement. Comparing the CTRL group to itself with the TOST showed that the threshold for non-equivalence was almost reached. This means that, given the number (n) of cells investigated, uncertainties from the method of assessment represent a limit for equivalence tests.

In contrast, all tested transmembrane currents were found to be equivalent. Current densities and kinetics of the K^+^ currents I_to_, I_K_, and I_K1_ were consistently unaltered after cold storage (Figs. 3 and 4). This contributed to the preservation of AP duration and shape as well as the stability of the resting membrane potential in all three groups. It also shows that, akin to the structural preservation of the TATS, transmembrane currents may generally remain much more stable in intact myocardium than in culture of isolated cells, where a significant decline of most currents was observed already after 24 h [1]. Although we did not record L-type Ca^2+^ currents (I_Ca,L_), it appears likely that there were no major changes in this current for the following reasons: First, AP shape and duration were not different after cold storage, which one would expect if I_Ca,L_, a major determinant of the AP plateau phase, was changed. Second, because I_Ca,L_ triggers intracellular Ca^2+^ release and excitation–contraction coupling, changes in I_Ca,L_ would also affect the amplitude and kinetics of the Ca^2+^ transient upstroke, which we did not observe here (Fig. 5). The direct evidence for the preservation of K^+^ currents as well as the indirect evidence for preservation of I_Ca,L_ is also in accordance with TATS integrity, because it was suggested that several important transmembrane currents exhibit particularly high densities within t-tubules [12, 33, 34]. Cell capacitance did not differ significantly after cold storage, also suggesting a preserved TATS. In summary, the results of this study allow the conclusion that there are no relevant differences in cardiomyocyte electrophysiological parameters after cold storage up to 24 h.

### Mitochondrial function

Cold storage over an extended period could affect mitochondria and, subsequently, metabolic status and energy supplies. Although we did not measure ATP/ADP ratio, we assessed mitochondrial health by the TMRM:MTG signal ratio, as suggested by others [4, 8, 15]. While the slightly lower volume density MTG in the CS group suggests overall fewer mitochondria or cell swelling, the portion of functional mitochondria can be expected to be comparable in the CS and CTRL group, given the even slightly higher TMRM signal intensity in CS (Fig. 7). These findings suggest that cardiomyocyte mitochondria are not or only mildly affected after 24 h of cold heart storage, such that at least the capacity to produce ATP via oxidative phosphorylation is preserved. Nevertheless, to what extend isolated cardiomyocytes rely on oxidative ATP production remains unclear, since most culture media contain high levels in glucose, which could be used for ATP production by anaerobic glycolysis.

### RNA expression

With only 128 differentially expressed genes, most of which were downregulated in the CSP group, we found generally very little evidence for altered mRNA expression after 24 h of preservation. The genes with the highest fold upregulation included unknown genes and ribosomal proteins and exhibited very little base mean expression in the CTRL (Supplemental Table 5), causing small absolute changes to appear as great fold changes. Gene set enrichment and pathway analyses revealed reduced expression of genes related to immune cells and inflammation (Supplemental Fig. 10), but not of genes related to any common cardiac or striated muscle ontology. Similarly, pathway analysis indicated mainly downregulation of interleukin-, immune response-, and inflammation-related signaling (Supplemental Fig. 11, Supplemental Table 6). It is possible that cold storage caused an inactivation or death of immune cells that were still present in the myocyte cell suspension. Alternatively, the 24 h period with cold temperature may have suppressed inflammatory pathways, such as NFkB signaling. However, all cardiac- or striated muscle-related pathways were unaffected. Also, we found no differentially expressed genes among those encoding for important ion channel subunits or proteins related to Ca^2+^ cycling, t-tubules, and excitation–contraction coupling (Table 1). This is in line with the structural and functional equivalence we found in the CSP and CS groups. We therefore conclude that cold storage can preserve gene expression, especially of those related to cardiomyocyte structure and function.

### Storage conditions

Some previous studies applying cold storage or perfusion reported functional decline and oxidative damage already after a few hours. Here, we report a high degree of preservation for 24 h. However, one study, for example, started with an initial perfusion at 37° C for 30–60 min before switching to a cooling solution at 6°−7° C, which was then maintained for 3 h [29]. Additionally, the perfusion solution was gassed with 95% O_2_ and 5% CO_2_ and did not contain BDM. Although we did not systematically alter the storage conditions, we strongly recommend placing the heart into cold storage solution immediately after removal to suppress excessive energy consumption under ischemic conditions. We also consider enrichment of the storage solution with oxygen unnecessary. In contrast, it could increase oxidative damage due to supraphysiological oxygen pressure. Finally, the addition of BDM may contribute to stopping spontaneous contractions and reducing ATP depletion, as BDM inhibits the myosin ATPase activity and has been shown to improve cardiac myocyte and tissue survival [7, 46].

### Proof of principle in a disease model

We successfully isolated cells from healthy and infarcted hearts that were shipped overnight from a different institution. This provides strong evidence that the suggested method can be applied for collaborations between laboratories separated by distances requiring overnight transport. It also provides evidence that the storage method is applicable to diseased hearts. Furthermore, we were able to detect significant structural and functional differences between the control and infarct group, demonstrating that important alterations in a disease model can be detected after 24 h of storage. Although the goal was not to characterize the disease model, the question arises why in the chosen example the cells from the infarcted hearts showed such a poor contractile function (Fig. 8). While our isolation protocol relies on coronary perfusion and thus most likely excludes the non-perfused infarct core, it is well established that cardiomyocyte function is significantly impaired even in the border and remote zones shortly after MI. Remote zones are not truly healthy, as they experience elevated wall stress, systemic inflammation, and neurohormonal activation, which can compromise cell health. Studies have shown that as early as 2 h post-MI, myocytes in the peri-infarct region exhibit reduced Ca^2^⁺ transient amplitude, abnormal Ca^2^⁺ waves, and impaired contractility [47], which persists at least until 1 week post-MI and also affects cells distant from the infarct[6].

### Limitations

A limitation of this work is that the number of cells after isolation was quantified by a manual method with relatively high technical variance. Although we did not observe major differences by visual inspection, it is possible that the number of cardiomyocytes after enzymatic digestion in the CTRL, CSP, and CS groups was not equivalent. However, the cell yield was high enough in all groups to carry out non-culture experiments without any restrictions. An interesting finding is that cell isolation via coronary perfusion was possible in the CS group, because one could expect that blood clots would have prevented enzyme access to the myocardium. Residual contractions of the heart after placing it in the *Cold storage solution*, however, may have caused squeezing of the coronary vessels, reducing the remaining blood. Enzyme solutions may also have gained access to the interstitial space via dilation and/or digestion of the vessel walls.

One conclusion that can be drawn from this study is that no particular haste is required after immersing the heart in the cold storage solution. However, although we did not investigate the effects of warm ischemia, earlier studies showed detrimental effects of warm ischemia on cardiomyocytes [24]. Thus, it still seems important to excise the heart quickly and put it immediately into the solution after the death of the animal to reduce the duration of warm ischemia to a minimum. Our tests of survivability in cell culture and TATS density revealed a slight reduction after 24 h in the cold storage group, but not after 6 h. Other parameters or behavior of isolated myocytes not tested here after culture could be altered when isolated after cold storage. However, since most experiments can be conducted within 6 h of isolation, the method appears acceptable for most research questions. Similarly, our results showed that after 48 h of storage, cell isolation and quality were severely compromised. Yet, 24 h is usually enough time to ship samples or hearts even between different countries within Europe.

We chose mouse, because this is the most widely used laboratory animal in cardiac research. The normal heart rate of mice is much higher than the pacing rates tested here. Thus, it cannot be excluded that at frequencies higher than 4 Hz differences in contractility or Ca^2+^ signaling would have occurred. Also, we cannot exclude that hearts or heart samples from other mammals respond differently to the presented preservation technique. However, because other studies have successfully implemented storage techniques for their specific experiments with rabbit [17, 45], pig [43], and human myocardium [14, 23], using similar storage solutions and periods, it seems likely that the method can be readily translated to other species.

### Summary and conclusion

The study shows that hearts can be stored for up to 24 h, even without prior perfusion, which will lead to greater flexibility in scheduling, reduction in the number of experimental animals, and facilitation of the exchange of samples between different institutions or laboratories without compromising data integrity. This will contribute to more efficient, ethical and collaborative cardiac research.

## Supplementary Information

Below is the link to the electronic supplementary material.

Supplementary file1 (DOCX 3155 KB)

Supplementary file2 (XLSX 7122 KB)

## Data Availability

The data presented in this study are available from the corresponding author upon reasonable request.
