# Supplementary material for: Cold storage of mouse hearts prior to cardiomyocyte isolation preserves electromechanical function, microstructure, and gene expression for 24 h
Source: Basic Res Cardiol. 2025 Jul 29;120(5):1055–74. doi: 10.1007/s00395-025-01131-y (PMC12518438; doi:10.1007/s00395-025-01131-y)
Supplement: Supplementary file 1 — Supplementary file1 (DOCX 3155 KB) [file 395_2025_1131_MOESM1_ESM.docx]

**Supplemental Material**

Cold storage of mouse hearts prior to cardiomyocyte isolation preserves electromechanical function, microstructure and gene expression for 24 h

Benedikt Pfeilschifter^1^, Aiora Martinez-Vilchez^1^, Zafar Iqbal^1^, Prapassorn Potue^1^, Dominik Fiegle^1^, Karoline Morhenn^2^, Alexander P. Schwoerer^2^, Tilmann Volk^1^, Thomas Seidel^1^

^1^ Institute of Cellular and Molecular Physiology, Friedrich-Alexander-University Erlangen-Nürnberg, Germany

^2^ Department of Cellular and Integrative Physiology, University Medical Centre Hamburg-Eppendorf, Hamburg, Germany

# Equations

## Equation 1: Determination of Conductivity and Boltzmann-fit for determining activation of I_to_

$$\frac{I_{\mathrm{Test}}}{(V_{\mathrm{Test}}-V_{\mathrm{Rev}})} =G$$

Where I_Test_ = current at given V_Test_, V_Test_ = applied test potential, V_Rev_ = Potassium reverse potential (-94 mV)

$$y=Bottom+ \frac{(Top-Bottom)}{1-\exp^{\left[ \frac{V_{50}-x}{\mathrm{Slope}} \right]}}$$

Where x is the applied test potential, V_50_ is the voltage where 50% is activated

## Equation 2: Boltzmann-fit for determining inactivation of I_to_

$$y=Bottom+ \frac{(Top-Bottom)}{1-\exp^{\left[ \frac{V_{50}-x}{\mathrm{Slope}} \right]}}$$

Where x is the applied test potential, V_50_ is the voltage where 50% is inactivated

## Equation 3: Two-phase association for fitting the time dependent recovery of I_to_

$$SpanFast=\left( Plateau-y_{0} \right)*\left( PercentFast*0.01 \right)$$

$$SpanSlow=\left( Plateau-Y_{0} \right)*\left( 100-PercentFast \right)*0.01$$

$$y= y_{0}+SpanFast\left[ 1-\exp^{({-K}_{\mathrm{fast}}*x)} \right]+SpanSlow\left[ 1-\exp^{(-K_{\mathrm{Slow}}*x)} \right]$$

## Equation 4: Two-phase decay for fitting I_to_ current traces

$$SpanFast=\left( y_{0}-Plateau \right)*\left( PercentFast*0.01 \right)$$

$$SpanSlow=\left( y_{0}-Plateau \right)*\left( 100-PercentFast \right)*0.01$$

$$y= Plateau+SpanFast*\left[ \exp^{({-K}_{\mathrm{fast}}*x)} \right]+SpanSlow*\left[ \exp^{(-K_{\mathrm{slow}}*x} \right]$$

# Supplemental Figure 1

| 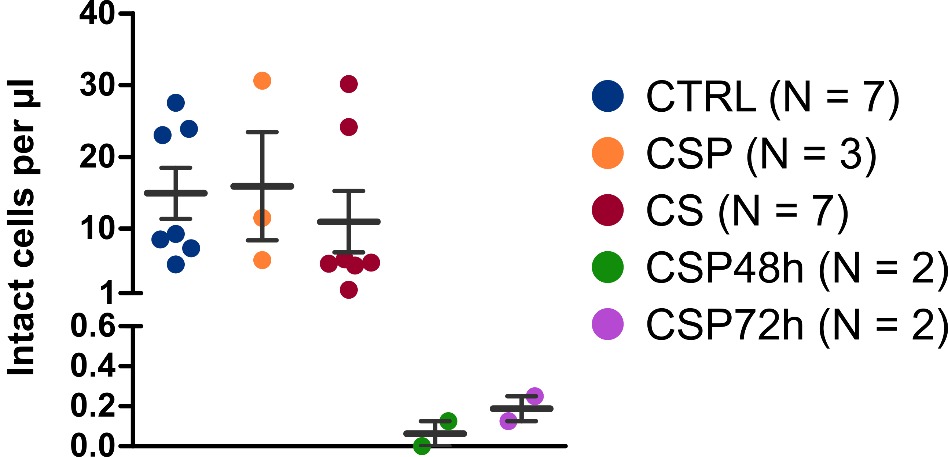 |
| --- |
| **Supplemental Figure 1: Cellular yield after isolation**  Number of intact cardiomyocytes from CTRL (N = 7 mice, blue), CSP (N = 3, orange), CS (N = 7, red), CSP48h (N = 2, green), CSP72h (N = 2, purple) determined with brightfield microscope and Neubauer chamber. Each data point represents the mean of five counting replicates per cell isolation. Statistical test used: unpaired, two-sided Welch’s t-test, p>0.05 between CTRL, CSP and CS. |

# Supplemental Figure 2

| 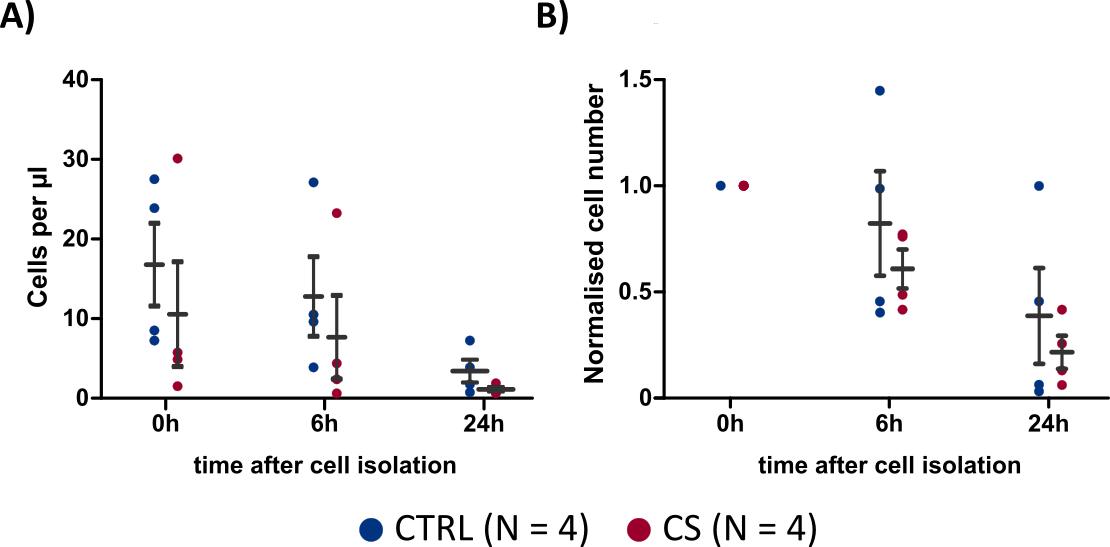 |
| --- |
| **Supplemental Figure 2: Survival in cell culture**  Cardiomyocytes from CTRL (blue, N = 4) and CS (red, N = 4) hearts were counted with brightfield microscope in a Neubauer chamber. Each data point represents the mean of five counting replicates. The timepoints indicate the time in culture after cell isolation. **A)** Absolute number of cells per µL. **B)** Relative number of cells, normalized to the number immediately after isolation (0 h). Statistical test used: unpaired, two-sided Welch’s t-test. p>0.05 between CTRL and CS in all comparisons |

# Supplemental Figure 3

| 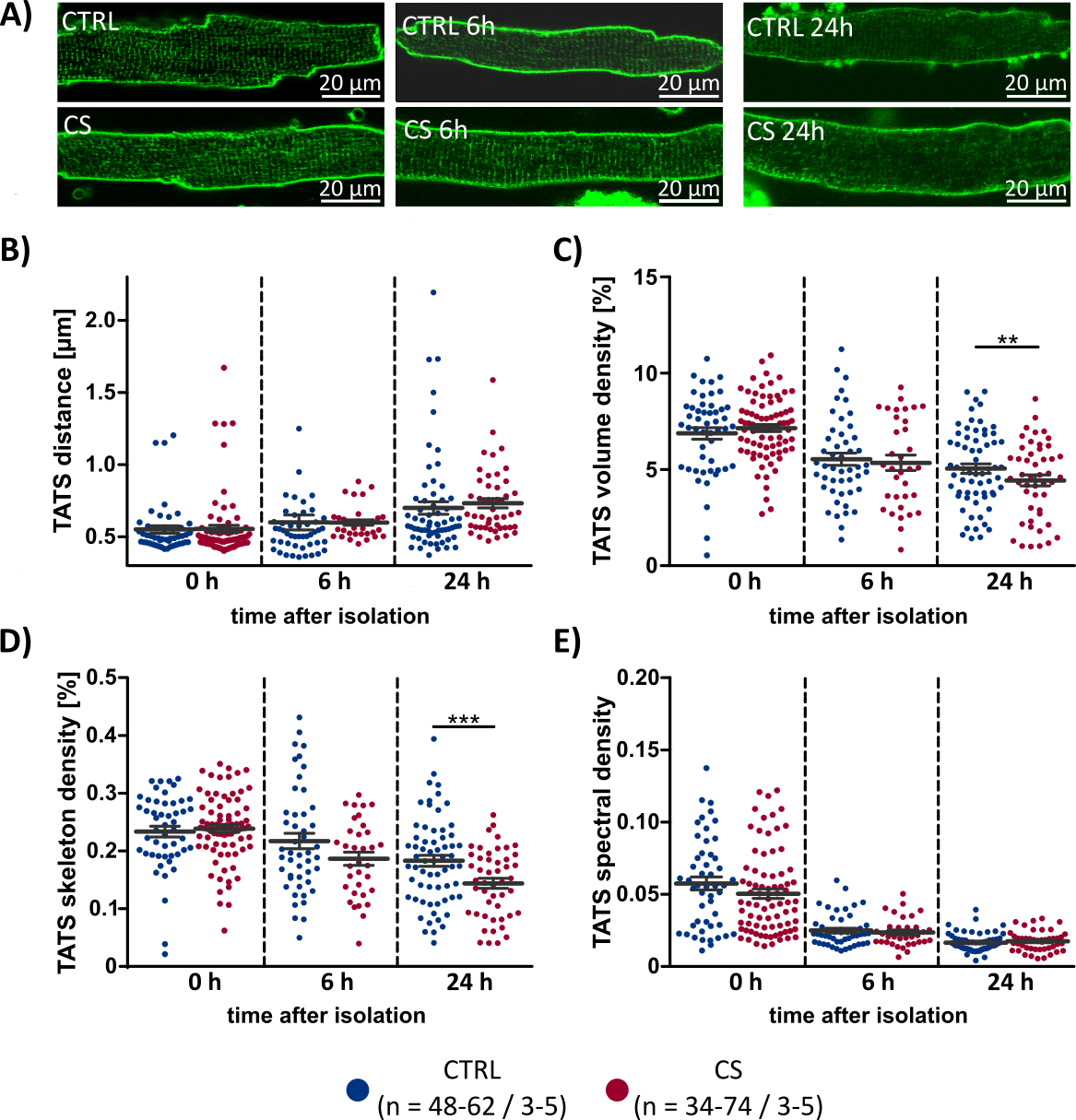 |
| --- |
| **Supplemental Figure 3: Changes of the TATS in cell culture**  Cardiomyocytes isolated from CTRL (blue, n = 48-62 / 3-5 cells/hearts) and CS hearts (red, n = 34-74 / 3-5) were stained with the membrane dye Di-8-ANEPPS and imaged with confocal microscopy to assess the transverse-axial tubular system (TATS) at the indicated times after isolation. **A)** Example images. **B)** Mean intracellular distance of transverse-axial tubules **C)** TATS volume density, **D)** TATS skeleton volume density. Densities are provided as the percentage of the cell volume occupied by the TATS or the TATS skeleton, respectively. **E)** TATS spectral density, a measure of t-tubule regularity. Statistical test used: unpaired, two-sided Welch’s t-test, * p<0.05, ** p<0.01, *** p<0.001 |

# Supplemental Figure 4

| 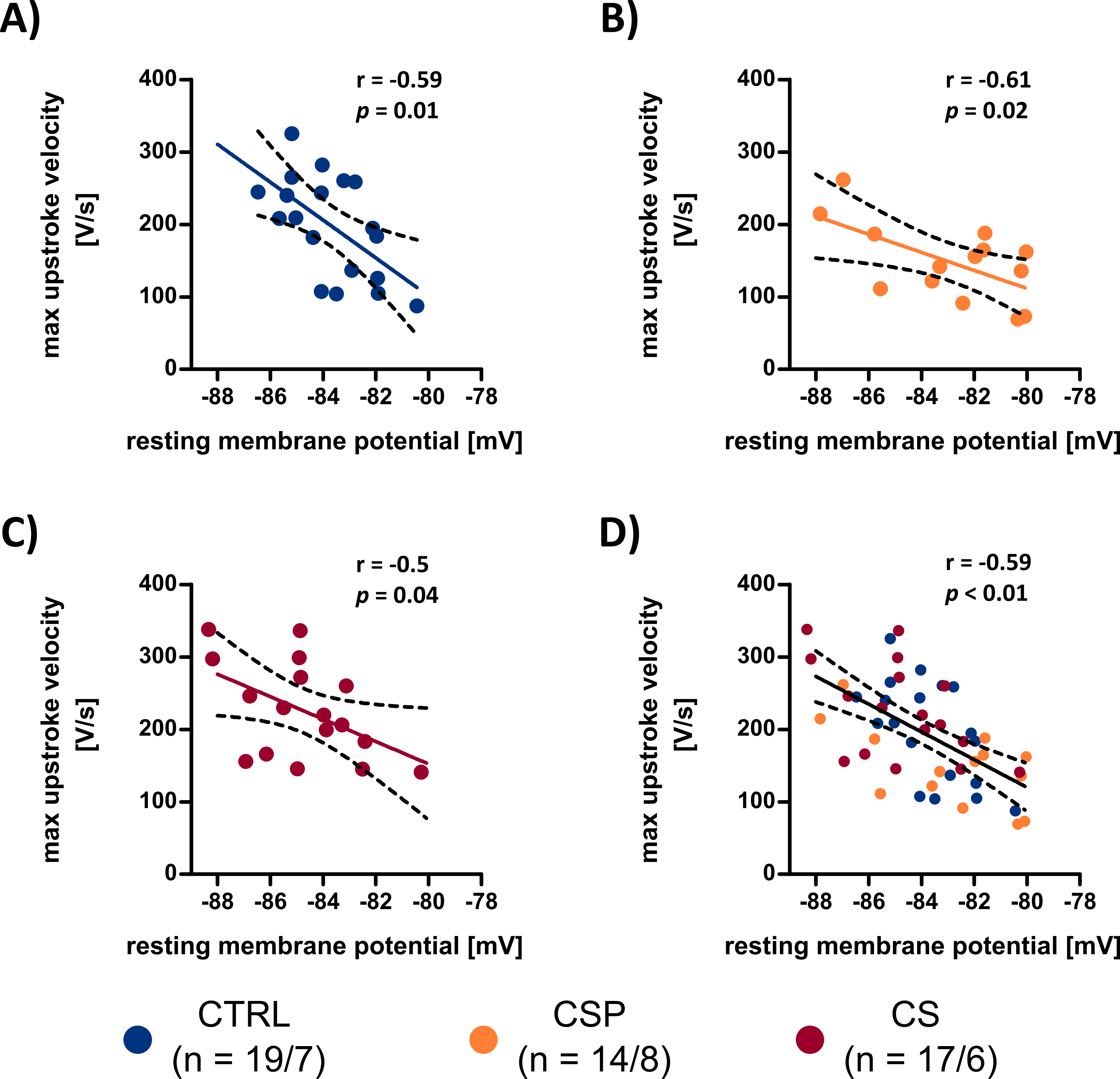 **Supplemental Figure 4: Correlation of maximum upstroke velocity with resting membrane potential**  Data points show the maximum upstroke velocity during the initial depolarisation phase of individual cardiomyocytes over the resting membrane potential with Pearson’s correlation coefficient *r* and the probability *p* that there is no correlation. Linear fit (solid lines) and 95% confidence intervals (dashed lines) are indicated. **A)** CTRL cells, blue, **B)** CSP cells, orange **C)** CS cells, red **D)** combination of all data points shown in A-C. Statistical test used: t-statistics vs constant model with zero slope. |
| --- |
|  |

# Supplemental Figure 5


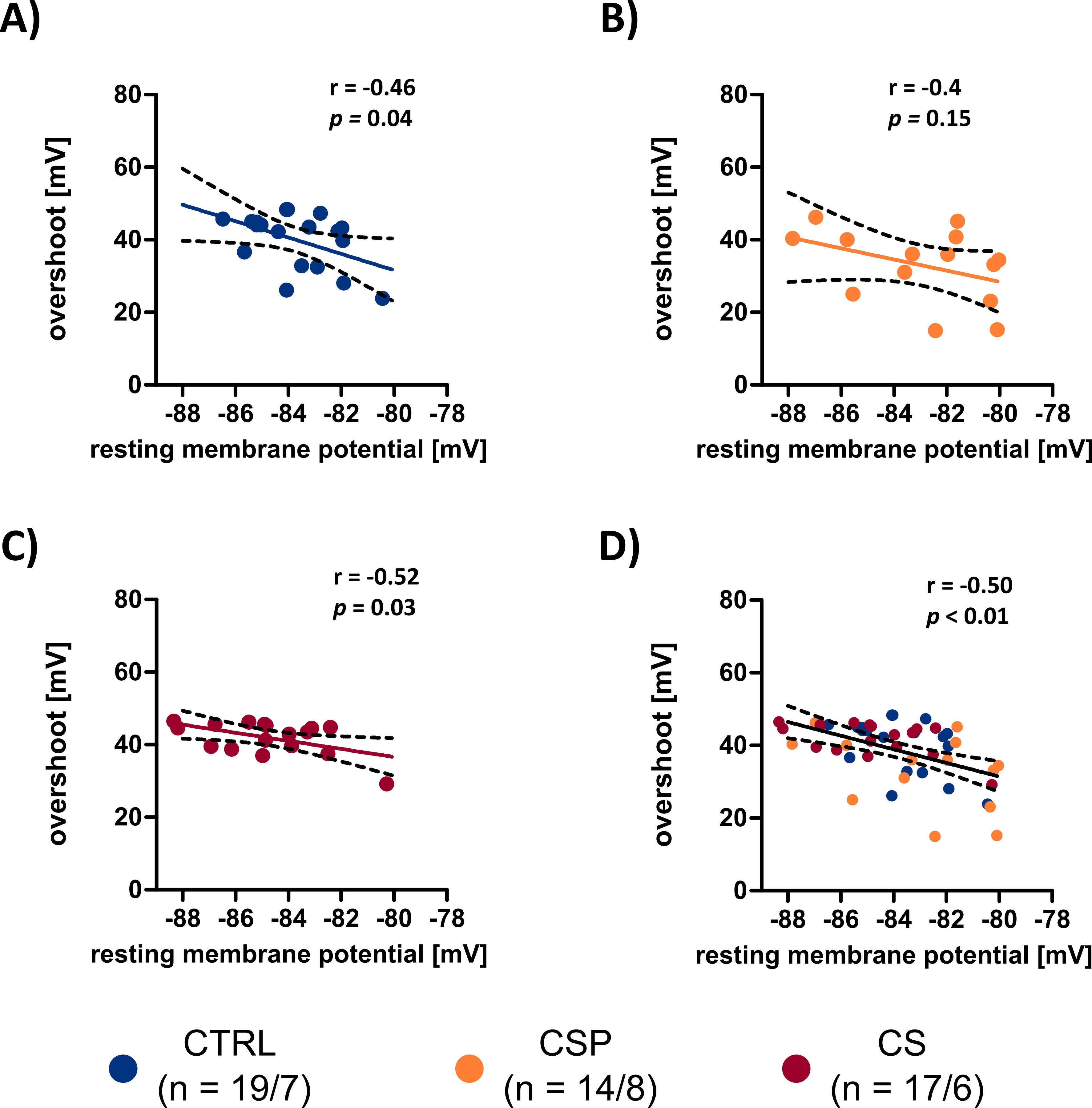


**Supplemental Figure 5: Correlation of action potential overshoot voltage with resting membrane potential**

Data points show the maximum upstroke velocity during the initial depolarisation phase of individual cardiomyocytes over the resting membrane potential with Pearson’s correlation coefficient *r* and the probability *p* that there is no correlation. Linear fit (solid lines) and 95% confidence intervals (dashed lines) are indicated. **A)** CTRL cells, blue **B)** CSP cells orange), **C)** CS cells red, **D)** combination of all data points shown in A-C. Statistical test used: t-statistics vs constant model with zero slope

# Supplemental Figure 6

**
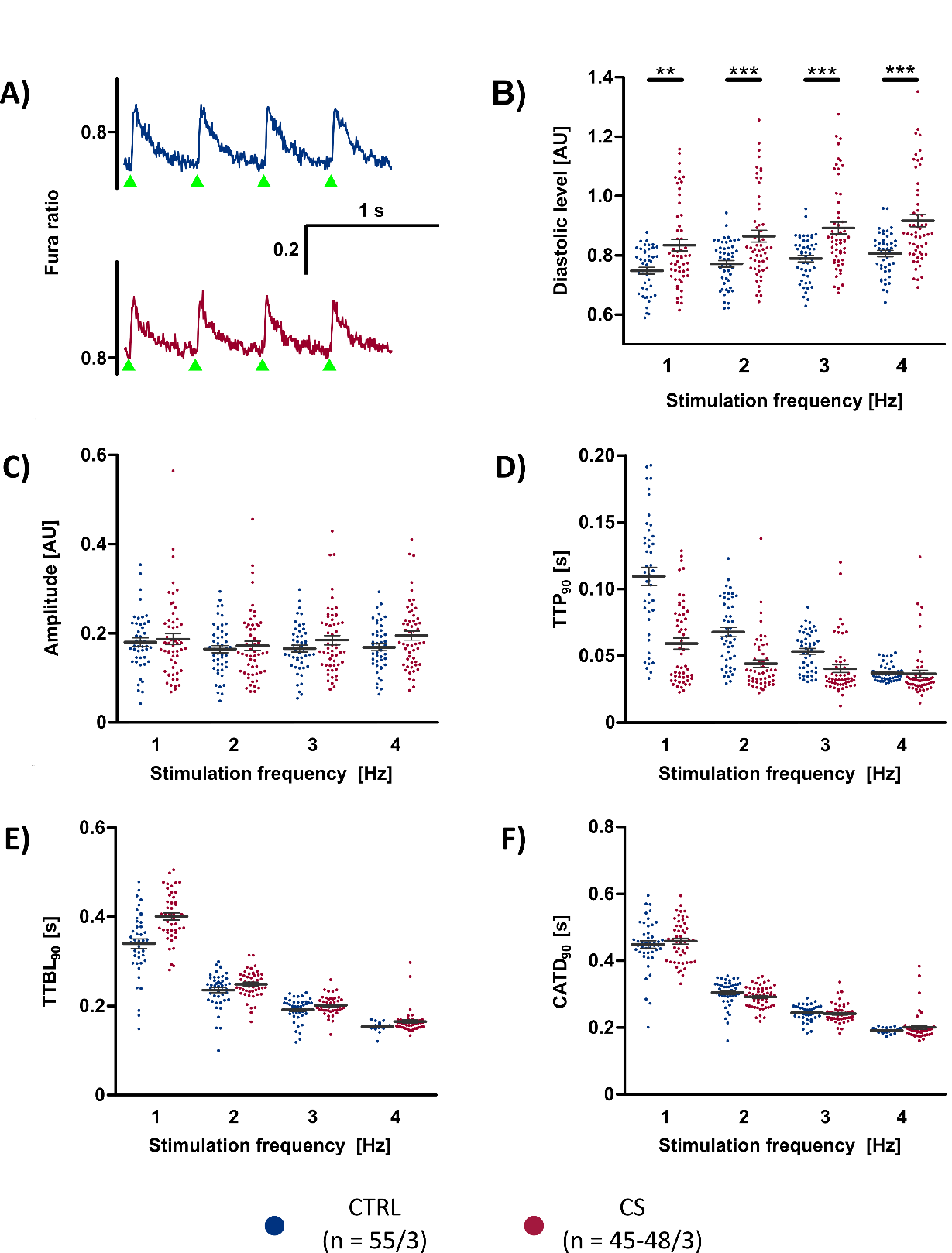
**

**Supplemental Figure 6: Intracellular Ca^2+^ recordings with Fura-2 at different pacing frequencies**

**A)** Representative recordings of the Fura-2 signal (emission ratio at excitation wavelengths 340nm and 380nm) at 2 Hz pacing of CTRL (blue, (n = 55/3 cells/hearts) and CS cells (red, n = 45-48/3). **B)** Diastolic Fura-2 signal at given pacing frequencies. **C)** Amplitude of Fura-2 signal upon electrical field stimulation at given frequencies **D)** Time to peak (TTP_90_), measured from 10% to the maximum of the signal during the upstroke phase of the Ca^2+^ transient. **E)** Time to baseline (TTBL_90_), measured from the maximum to 10% of the signal during the decay phase of the Ca^2+^ transient. **F)** Ca^2+^ transient duration, measured from 10% of the signal during the upstroke phase until 10% of the signal during the decay phase (CATD_90_ = TTP_90_ + TTBL_90_). Statistical test used: unpaired, two-sided Welch’s t-test, * p<0.05, ** p<0.01, *** p<0.001.

# Supplemental Figure 7

**
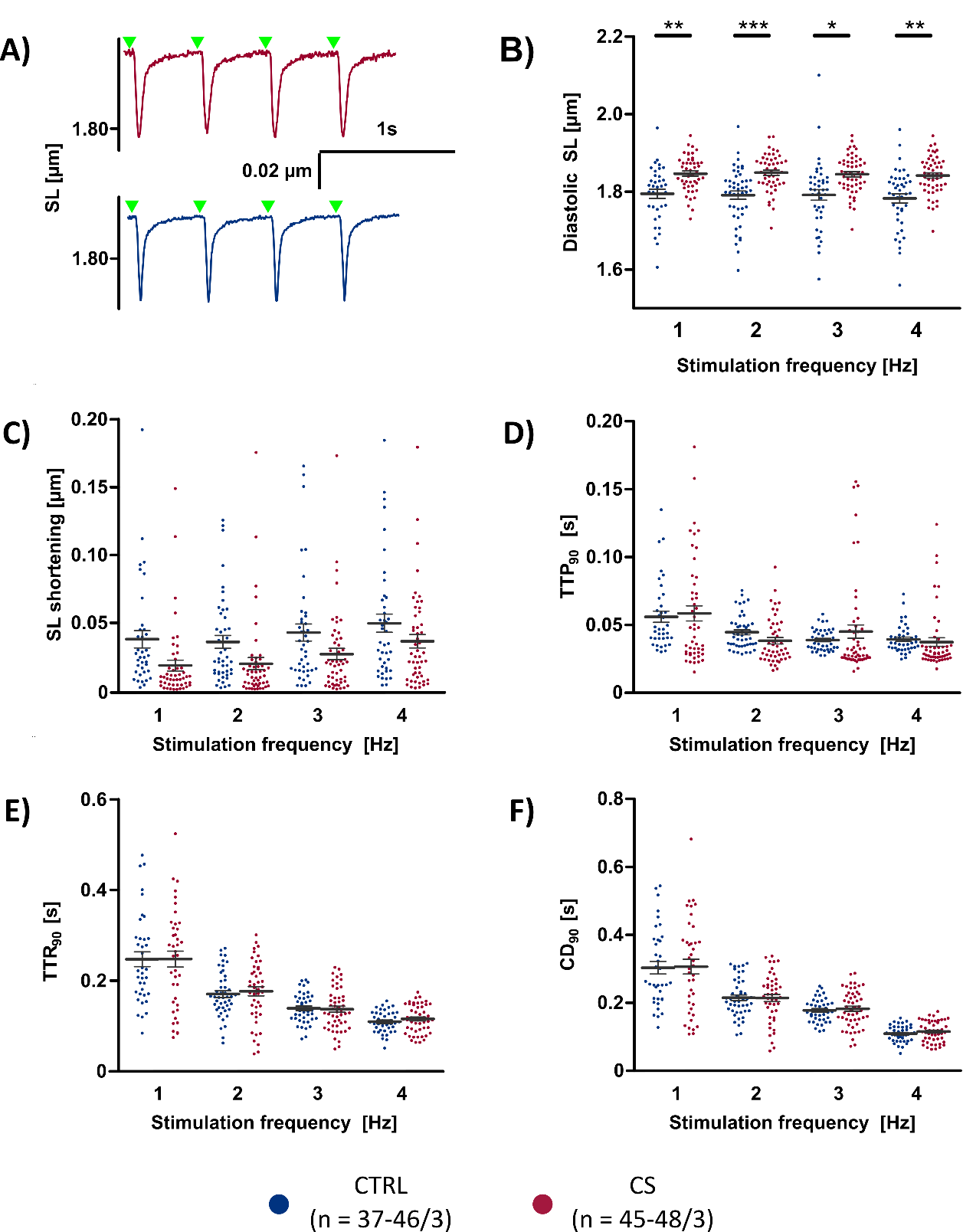
**

**Supplemental Figure 7: Contraction Parameters**

**A)** Representative examples of sarcomere length (SL) changes upon electrical stimulation at 2 Hz in the CTRL (blue, n = 37-46/3 cells/hearts) and CS group (red, n = 45-48/3), determined by Fourier analysis of striations visible in bright-field microscopy. Time of stimulation is indicated by green triangles. **B)** Diastolic SL at different pacing frequencies. **C)** Maximum SL shortening at different pacing frequencies. **D)** Time to peak (TTP_90_), measured from 10% to the maximum of the contraction **E)** Time to relaxation (TTR_90_), measured from the point of maximum shortening to 10% of the diastolic SL during relaxation **F)** Contraction duration, measured from 10% of the shortening during the contraction phase until 10% of the shortening during the relaxation phase (CD_90_ = TTP_90_ + TTR_90_). Statistical test used: unpaired, two-sided Welch’s t-test, * p<0.05, ** p<0.01, *** p<0.001.

# Supplemental Figure 8

**
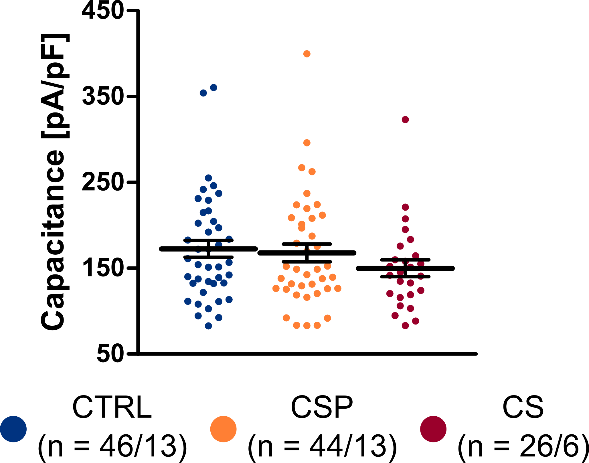
**

**Supplemental Figure 8: Cell Capacitance**

The capacitance of isolated cardiomyocytes in the CTRL (blue, n = 46 cells/13 hearts), CSP (orange, n = 44/13) and CS (red, n = 26/6) groups was measured during patch clamp experiments. Statistical test used: unpaired, two-sided Welch’s t-test, * p<0.05, ** p<0.01, *** p<0.001.

# Supplemental Figure 9


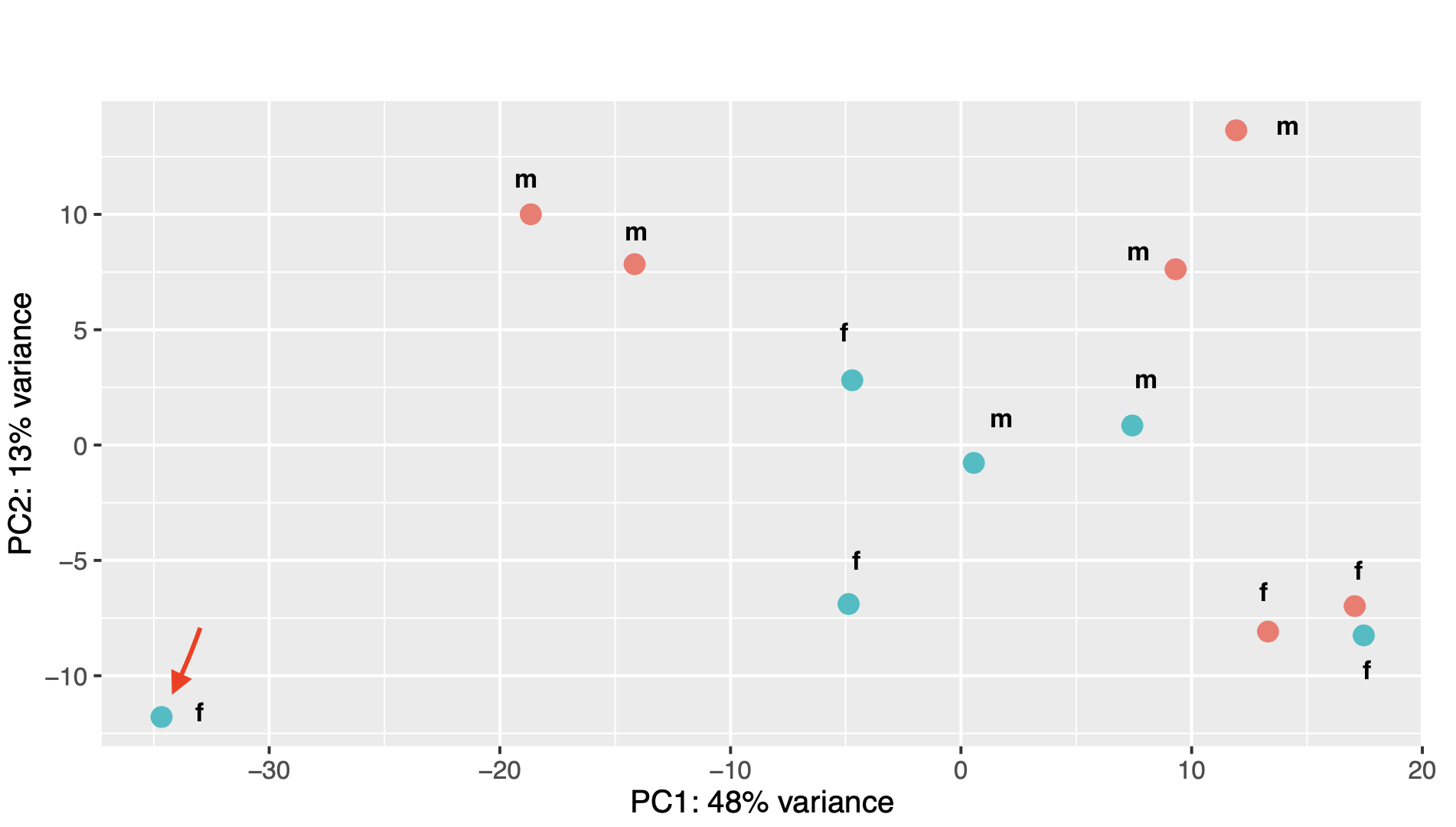


**Supplemental Figure 9: Principal component analysis of RNAseq data**

Mint-coloured dots indicate samples from the CTRL group, orange dots indicate samples from the CTRL. Sex is indicated by black letters (m, male; f, female). One sample (arrow) was identified as outlier and excluded from further analysis.

# Supplemental Figure 10


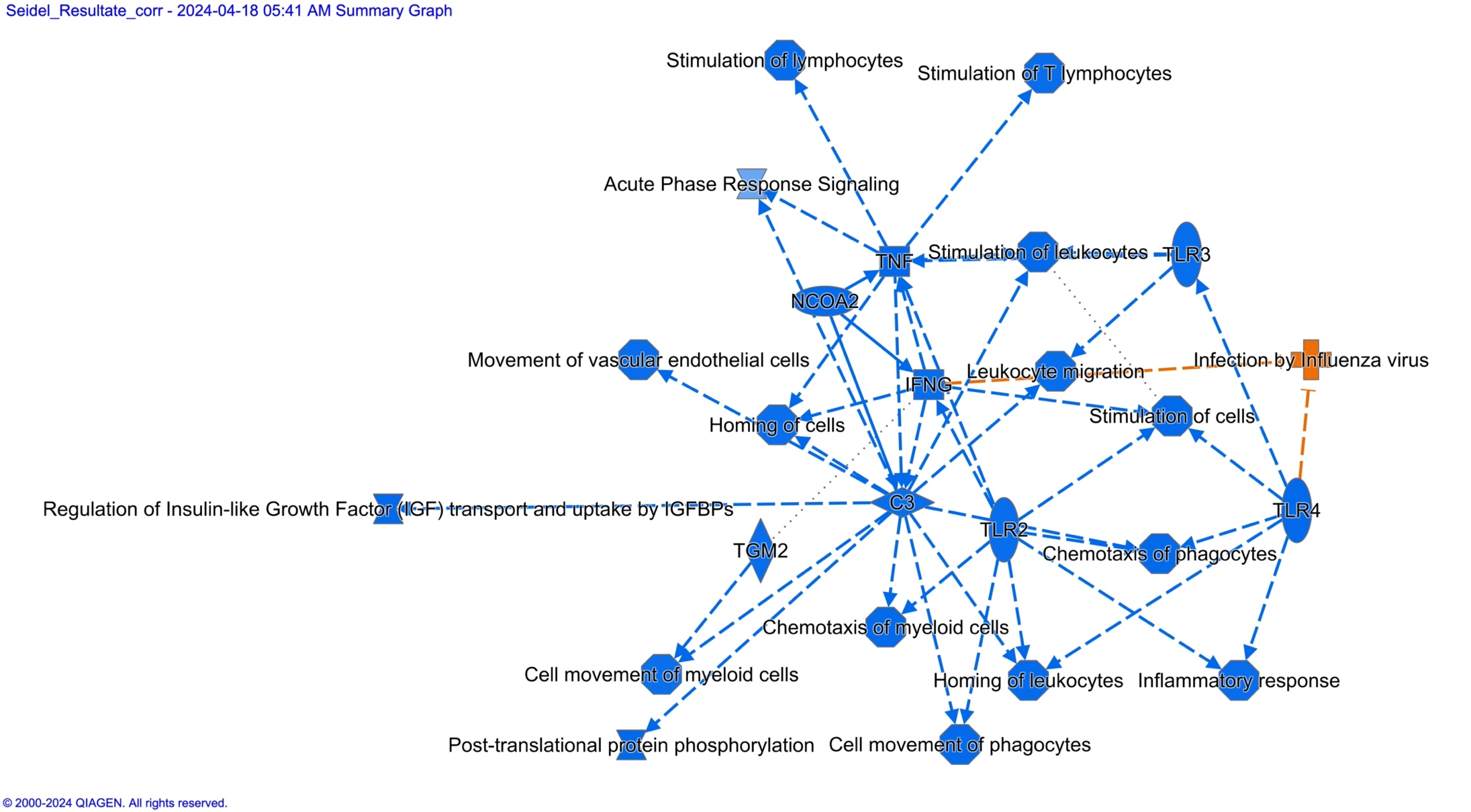


**Supplemental Figure 10: Gene set enrichment analysis of differentially expressed genes from RNASeq in CSP vs CTRL.**

The downregulated pathways (blue) comprised mainly pathways and signals that are relevant for immune cells and inflammation. The upregulated pathway (orange) was also not specific for cardiomyocytes.

# Supplemental Figure 11


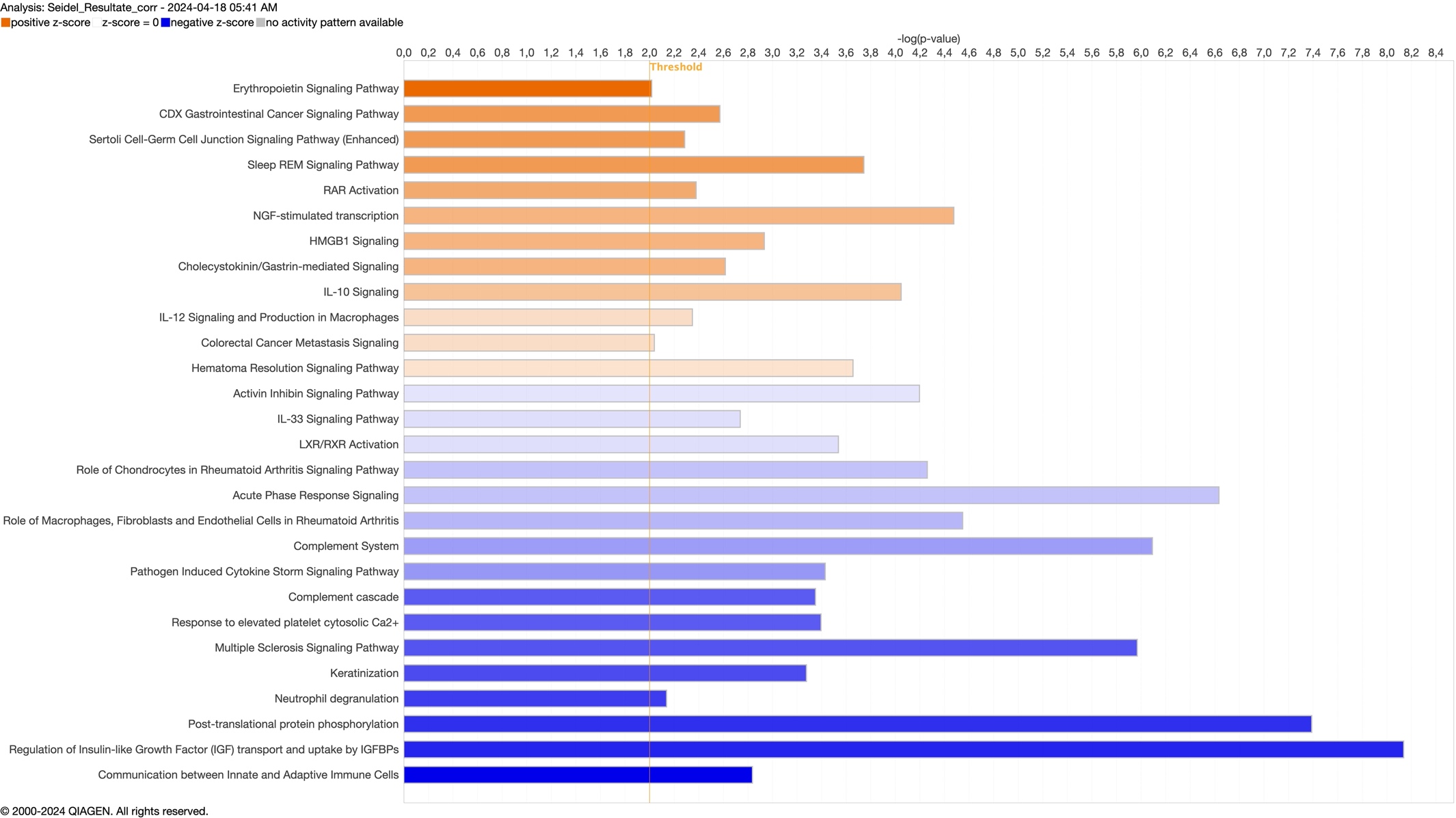


**Supplemental Figure 11: Pathway analysis of differentially expressed genes from RNASeq in CSP vs CTRL.**

The significantly (p<0.01) upregulated (orange) and downregulated (blue) genes belonged to pathways and signals relevant for immune cells and inflammation. Genes belonging to pathways and signals considered relevant for cardiac hypertrophy or disease were not significantly altered.

# Supplemental Figure 12


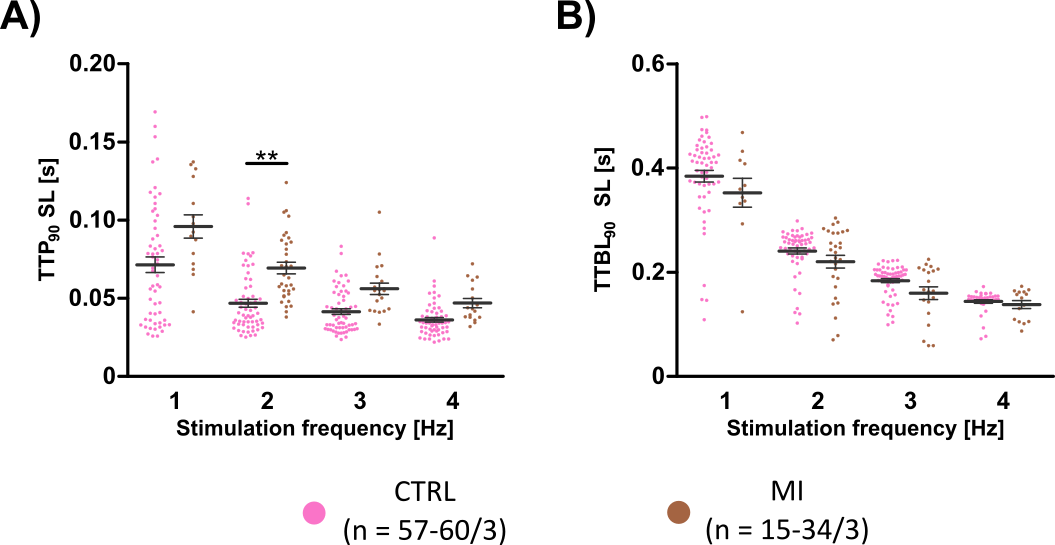


**Supplemental Figure 12: Kinetics of intracellular Ca^2+^ transients**

Kinetic parameters of CTRL (pink, n = 57-60/3 cells/hearts) and surgically infarcted MI hearts (n = 15-34/3, brown). **A)** Time to peak (TTP_90_), measured from 10% to the maximum of the signal during the upstroke phase of the Ca^2+^ transient. **B)** Time to baseline (TTBL_90_), measured from the maximum to 10% of the signal during the decay phase of the Ca^2+^ transient. Statistical test used: unpaired, two-sided Welch’s t-test, ** p<0.01.

# Supplemental Table 1: Troponin T content of hearts

| **Animal** | **Heart weight** | **TnT measured**  **in 1:50 diluted buffer** | **TnT conc in 40 ml homogenisation buffer** | **Total TnT per heart** |
| --- | --- | --- | --- | --- |
| 1 | 150,6 mg | 0.259 µg/l | 12.95 µg/l | 0.518 µg |
| 2 | 155,4 mg | 0.208 µg/l | 10.4 µg/l | 0.416 µg |
| 3 | 126,2 mg | 0.162 µg/l | 8.1 µg/l | 0.324 µg |
| 4 | 117.2 mg | 0.133 µg/l | 6.65 µg/l | 0.266 µg |
| 5 | 155.4 mg | 0.113 µg/l | 5.56 µg/l | 0.222 µg |

**Supplemental Table 1.** Troponin T (TnT) measurements of homogenized hearts to estimate total Troponin T amount per heart as a reference to Troponin T found in the storage solutions.

# Supplemental Table 2: Confidence intervals of mean differences (TATS and electrophysiologic parameters)

| **Parameter** | **Unit** | **CTRL vs CTRL** | | **CSP vs CTRL** | | **CS vs CTRL** | |
| --- | --- | --- | --- | --- | --- | --- | --- |
|  |  | ∆ (absolute) | ∆ (relative) | ∆ (absolute) | ∆ (relative) | ∆ (absolute) | ∆ (relative) |
| TATS distance | µm | 0.069 | 0.11 | 0.076 | 0.12 | 0.076 | 0.12 |
| TATS volume density | % | 0.75 | 0.11 | 0.95 | 0.14 | 0.88 | 0.13 |
| TATS skeleton density | % | 0.023 | 0.1 | 0.026 | 0.11 | 0.026 | 0.11 |
| TATS spectral density | - | 0.011 | 0.19 | 0.015 | 0.27 | 0.016 | 0.29 |
|  |  |  |  |  |  |  |  |
| TATS distance after 6h | µm | 0.096 | 0.16 |  |  | 0.126 | 0.21 |
| TATS volume density after 6h | % | 0.96 | 0.14 |  |  | 1.31 | 0.19 |
| TATS skeleton density after 6h | % | 0.035 | 0.15 |  |  | 0.65 | 0.28 |
| TATS spectral density after 6h | - | 0.003 | 0.16 |  |  | 0.005 | 0.22 |
|  |  |  |  |  |  |  |  |
| TATS distance after 24h |  | 0.107 | 0.15 |  |  | 0.129 | 0.18 |
| TATS volume density after 24h |  | 0.6 | 0.12 |  |  | 1.26 | 0.25 |
| TATS skeleton density after 24h |  | 0.024 | 0.13 |  |  | 0.061 | **0.33** |
| TATS spectral density after 24h |  | 0.0019 | 0.12 |  |  | 0.0017 | 0.19 |
|  |  |  |  |  |  |  |  |
| Capacitance | pF | 24.16 | 0.14 | 29.36 | 0.17 | 46.6 | 0.27 |
| RMP | mV | 1.67 | 0.02 | 2.51 | 0.03 | 2.51 | 0.03 |
| AP amplitude | mV | 4.94 | 0.04 | 14.84 | 0.04 | 7.42 | 0.06 |
| dV/dt_max_ | V/s | 43.57 | 0.22 | 87.14 | **0.44** | 67.34 | **0.34** |
|  |  |  |  |  |  |  |  |
| overshoot | mV | 4.4 | 0.11 | 12.79 | **0.32** | 5.6 | 0.14 |
| APD_0mV_ | ms | 0.92 | 0.22 | 2.65 | **0.63** | 1.26 | 0.3 |
| APD_20_ | ms | 0.5 | 0.18 | 0.95 | **0.34** | 0.75 | 0.27 |
| APD_50_ | ms | 2.44 | 0.29 | 5.82 | **0.69** | 3.46 | **0.41** |
| APD_90_ | ms | 17.46 | 0.26 | 27.54 | **0.41** | 27.54 | **0.41** |
|  |  |  |  |  |  |  |  |
| I_K_ at +60 mV | pA/pF | 2.64 | 0.14 | 2.64 | 0.14 | 3.77 | 0.2 |
|  |  |  |  |  |  |  |  |
| I_to_ at +60 mV | pA/pF | 7.18 | 0.17 | 8.45 | 0.2 | 12.25 | 0.29 |
| I_to_ 50% activation voltage | mV | 1.5 | 0.1 | 3.16 | 0.21 | 1.8 | 0.12 |
| I_to_ activation slope | mV | 0.66 | 0.04 | 1.15 | 0.07 | 0.82 | 0.05 |
| I_to_ 50% inactivation voltage | mV | 2.04 | 0.06 | 2.72 | 0.08 | 1.7 | 0.05 |
| I_to_ inactivation slope | mV | 0.37 | 0.07 | 0.85 | 0.16 | 0.64 | 0.12 |
| I_to_ fast recovery time constant (τ fast) | ms | 10.17 | 0.2 | 15.76 | **0.31** | 20.84 | **0.46** |
| I_to_ slow recovery time constant (τ slow) | ms | 422.12 | 0.32 | 593.61 | **0.45** | 685.95 | **0.52** |
| I_to_ fast decay time constant (τ fast) | ms | 5.08 | 0.1 | 9.15 | 0.18 | 6.1 | 0.12 |
| I_to_ slow decay time constant (τ slow) | ms | 224.25 | 0.17 | 329.78 | 0.25 | 474.89 | **0.36** |
|  |  |  |  |  |  |  |  |
| I_K1_ at -120 mV | pA/pF | 1.11 | 0.19 | 1.7 | 0.29 | 1.52 | 0.26 |
|  |  |  |  |  |  |  |  |
| Pearson corr. TMRM/MTG | - | 0.035 | 0.05 |  |  | 0.042 | 0.06 |
| MTG volume density | % | 2.736 | 0.09 |  |  | 6.08 | 0.2 |
| MTG Intensity | - | 3.32 | 0.17 |  |  | 7.22 | **0.37** |
| TMRM Intensity | - | 5.86 | 0.18 |  |  | 15.3 | **0.47** |
| TMRM/MTG Intensity Ratio | - | 0.27 | 0.15 |  |  | 1.24 | **0.67** |

**Supplemental Table 2.** Relative (fraction of CTRL) and absolute intervals (± ∆) of group differences, obtained with a two one sided t-test (TOST), using an alpha of 0.05. Interpret as follows: if, for example, the absolute ∆ = 1 ms and relative ∆ = 0.15, then the observed means (μ_CTRL_ and μ_CS_) and variances indicate with a 95% certainty that the true difference (μ_CS_ - μ_CTRL_) falls into the interval ±1 ms, which corresponds to 15% of μ_CTRL_. Thus, if an effect size of ≤ 1 ms is considered irrelevant, the two groups can be considered equivalent with a certainty of 95%. CTRL vs CTRL reflects the technical uncertainty stemming from the method of assessment. Bold numbers (>0.3) indicate non-equivalence.

# Supplemental Table 3: Confidence intervals of mean differences (Ca^2+^ signal parameters)

| **Parameter** | **Unit** | **CTRL vs CTRL** | | **CSP vs CTRL** | |
| --- | --- | --- | --- | --- | --- |
|  |  | ∆ (absolute) | ∆ (relative) | ∆ (absolute) | ∆ (relative) |
| Fura diastolic level |  |  |  |  |  |
| 1 Hz | / | 0.036 | 0.04 | 0.063 | 0.07 |
| 2 Hz | / | 0.037 | 0.04 | 0.064 | 0.07 |
| 3 Hz | / | 0.005 | 0.04 | 0.008 | 0.06 |
| 4 Hz | / | 0.029 | 0.03 | 0.058 | 0.06 |
|  |  |  |  |  |  |
| Fura amplitude |  |  |  |  |  |
| 1 Hz | / | 0.02 | 0.14 | 0.04 | 0.26 |
| 2 Hz | / | 0.017 | 0.14 | 0.04 | **0.33** |
| 3 Hz | / | 0.016 | 0.13 | 0.046 | **0.36** |
| 4 Hz | / | 0.017 | 0.13 | 0.044 | **0.35** |
|  |  |  |  |  |  |
| Fura TTP90 |  |  |  |  |  |
| 1 Hz | ms | 11.17 | 0.12 | 14.89 | 0.16 |
| 2 Hz | ms | 6.0 | 0.1 | 6.0 | 0.1 |
| 3 Hz | ms | 3.71 | 0.08 | 5.1 | 0.11 |
| 4 Hz | ms | 2.37 | 0.06 | 3.56 | 0.09 |
|  |  |  |  |  |  |
| Fura TTBL90 |  |  |  |  |  |
| 1 Hz | ms | 22.37 | 0.06 | 33.55 | 0.09 |
| 2 Hz | ms | 14.26 | 0.06 | 21.38 | 0.09 |
| 3 Hz | ms | 9.56 | 0.05 | 11.48 | 0.06 |
| 4 Hz | ms | 4.52 | 0.03 | 12 | 0.08 |
|  |  |  |  |  |  |
| Fura CATD90 |  |  |  |  |  |
| 1 Hz | ms | 18.64 | 0.04 | 27.96 | 0.06 |
| 2 Hz | ms | 11.87 | 0.04 | 20.77 | 0.07 |
| 3 Hz | ms | 7.13 | 0.03 | 1.2 | 0.05 |
| 4 Hz | ms | 3.8 | 0.02 | 7.61 | 0.04 |

**Supplemental Table 3.** Relative (fraction of CTRL) and absolute intervals (± ∆) of group differences, obtained with a two one sided t-test (TOST), using an alpha of 0.05. Interpret as follows: if, for example, the absolute ∆ = 1 ms and relative ∆ = 0.15, then the observed means (μ_CTRL_ and μ_CSP_) and variances indicate with a 95% certainty that the true difference (μ_CSP_ - μ_CTRL_) falls into the interval ±1 ms, which corresponds to 15% of μ_CTRL_. Thus, if an effect size of ≤ 1 ms is considered irrelevant, the two groups can be considered equivalent with a certainty of 95%. CTRL vs CTRL reflects the technical uncertainty stemming from the method of assessment. Bold numbers (>0.3) indicate non-equivalence.

# Supplemental Table 4: Confidence intervals of mean differences (contraction parameters)

| **Parameter** | **Unit** | **CTRL vs CTRL** | | **CTRL vs CSP** | |
| --- | --- | --- | --- | --- | --- |
|  |  | ∆ (absolute) | ∆ (relative) | ∆ (absolute) | ∆ (relative) |
| diastolic sarcomere length |  |  |  |  |  |
| 1 Hz | µm | 0.018 | 0.01 | 0.018 | 0.01 |
| 2 Hz | µm | 0.018 | 0.01 | 0.018 | 0.01 |
| 3 Hz | µm | 0.018 | 0.01 | 0.018 | 0.01 |
| 4 Hz | µm | 0.018 | 0.01 | 0.018 | 0.02 |
|  |  |  |  |  |  |
| sarcomere shortening |  |  |  |  |  |
| 1 Hz | µm | 0.0086 | 0.35 | 0.013 | **0.54** |
| 2 Hz | µm | 0.0052 | 0.29 | 0.016 | **0.9** |
| 3 Hz | µm | 0.0061 | 0.25 | 0.018 | **0.75** |
| 4 Hz | µm | 0.0074 | 0.24 | 0.023 | **0.64** |
|  |  |  |  |  |  |
| TTP90 |  |  |  |  |  |
| 1 Hz | ms | 12.46 | 0.19 | 14.43 | 0.22 |
| 2 Hz | ms | 5.2 | 0.11 | 7.56 | 0.16 |
| 3 Hz | ms | 3.44 | 0.09 | 6.88 | 0.18 |
| 4 Hz | ms | 3.21 | 0.09 | 7.84 | 0.22 |
|  |  |  |  |  |  |
| TTR90 |  |  |  |  |  |
| 1 Hz | ms | 35.28 | 0.14 | 45.37 | 0.18 |
| 2 Hz | ms | 21.17 | 0.11 | 24 | 0.12 |
| 3 Hz | ms | 14.61 | 0.1 | 17.53 | 0.12 |
| 4 Hz | ms | 11 | 0.1 | 15.39 | 0.14 |
|  |  |  |  |  |  |
| CD90 |  |  |  |  |  |
| 1 Hz | ms | 41.37 | 0.13 | 44.55 | 0.14 |
| 2 Hz | ms | 21.57 | 0.09 | 21.57 | 0.09 |
| 3 Hz | ms | 16.59 | 0.09 | 22.12 | 0.12 |
| 4 Hz | ms | 11.66 | 0.08 | 13.11 | 0.09 |

**Supplemental Table 4.** Relative (fraction of CTRL) and absolute intervals (± ∆) of group differences, obtained with a two one sided t-test (TOST), using an alpha of 0.05. Interpret as follows: if, for example, the absolute ∆ = 1 ms and relative ∆ = 0.15, then the observed means (μ_CTRL_ and μ_CSP_) and variances indicate with a 95% certainty that the true difference (μ_CSP_ - μ_CTRL_) falls into the interval ±1 ms, which corresponds to 15% of μ_CTRL_. Thus, if an effect size of ≤ 1 ms is considered irrelevant, the two groups can be considered equivalent with a certainty of 95%. CTRL vs CTRL reflects the technical uncertainty stemming from the method of assessment. Bold numbers (>0.3) indicate non-equivalence.

# Supplemental Table 5. Significantly differentially expressed genes in CSP

| Ensembl 102  Gene stable ID | Avg  raw  counts | log2Fold  Change | p adjusted | Name | Description |
| --- | --- | --- | --- | --- | --- |
| ENSMUSG00000070960 | 8.2 | 30.00 | 2.40E-12 | Gm19680 | predicted gene, 19680 |
| ENSMUSG00000078087 | 51.9 | 23.86 | 2.87E-06 | Rps12l1 | ribosomal protein S12-like 1 |
| ENSMUSG00000086922 | 42.4 | 23.41 | 3.82E-06 | Gm13835 | predicted gene 13835 |
| ENSMUSG00000089739 | 24.6 | 15.09 | 2.78E-02 | Gm20431 | predicted gene 20431 |
| ENSMUSG00000058626 | 14.6 | 4.98 | 2.04E-02 | Capn11 | calpain 11 |
| ENSMUSG00000034227 | 16.3 | 3.50 | 1.85E-02 | Foxj1 | forkhead box J1 |
| ENSMUSG00000081738 | 28.9 | 2.55 | 2.13E-02 | Hmgb1-ps2 | high mobility group box 1, pseudogene 2 |
| ENSMUSG00000003545 | 22,037.4 | 1.86 | 4.37E-20 | Fosb | FBJ osteosarcoma oncogene B |
| ENSMUSG00000019960 | 2,515.6 | 1.72 | 1.50E-04 | Dusp6 | dual specificity phosphatase 6 |
| ENSMUSG00000097296 | 139.9 | 1.55 | 2.47E-03 | Gm26532 | predicted gene, 26532 |
| ENSMUSG00000024190 | 16,234.3 | 1.15 | 5.77E-08 | Dusp1 | dual specificity phosphatase 1 |
| ENSMUSG00000037001 | 147.5 | 1.14 | 3.89E-03 | Zfp39 | zinc finger protein 39 |
| ENSMUSG00000052684 | 38,606.3 | 1.05 | 1.16E-06 | Jun | jun proto-oncogene |
| ENSMUSG00000022602 | 703.7 | 1.04 | 5.62E-03 | Arc | activity regulated cytoskeletal-associated protein |
| ENSMUSG00000095253 | 326.3 | 1.02 | 1.46E-02 | Zfp799 | zinc finger protein 799 |
| ENSMUSG00000028410 | 4,188.0 | 0.67 | 3.46E-03 | Dnaja1 | DnaJ heat shock protein family (Hsp40) member A1 |
| ENSMUSG00000021250 | 43,749.1 | 0.64 | 1.31E-03 | Fos | FBJ osteosarcoma oncogene |
| ENSMUSG00000032531 | 3,721.7 | 0.64 | 2.07E-02 | Amotl2 | angiomotin-like 2 |
| ENSMUSG00000054364 | 14,385.3 | 0.59 | 4.53E-03 | Rhob | ras homolog family member B |
| ENSMUSG00000039899 | 3,151.2 | -0.78 | 7.04E-04 | Fgl2 | fibrinogen-like protein 2 |
| ENSMUSG00000009633 | 2,076.2 | -0.78 | 9.22E-03 | G0s2 | G0/G1 switch gene 2 |
| ENSMUSG00000022893 | 7,606.2 | -0.82 | 4.70E-05 | Adamts1 | a disintegrin-like and metallopeptidase (reprolysin type) with thrombospondin type 1 motif, 1 |
| ENSMUSG00000008035 | 5,864.7 | -0.94 | 2.45E-04 | Mid1ip1 | Mid1 interacting protein 1 (gastrulation specific G12-like (zebrafish)) |
| ENSMUSG00000026185 | 8,784.5 | -0.95 | 7.58E-08 | Igfbp5 | insulin-like growth factor binding protein 5 |
| ENSMUSG00000029135 | 4,028.7 | -0.98 | 3.64E-05 | Fosl2 | fos-like antigen 2 |
| ENSMUSG00000036098 | 304.9 | -1.01 | 1.86E-02 | Myrf | myelin regulatory factor |
| ENSMUSG00000058297 | 539.0 | -1.13 | 2.06E-05 | Spock2 | sparc/osteonectin, cwcv and kazal-like domains proteoglycan 2 |
| ENSMUSG00000039457 | 215.1 | -1.14 | 1.44E-02 | Ppl | periplakin |
| ENSMUSG00000030103 | 5,667.4 | -1.15 | 1.60E-07 | Bhlhe40 | basic helix-loop-helix family, member e40 |
| ENSMUSG00000037465 | 1,297.0 | -1.17 | 4.92E-02 | Klf10 | Kruppel-like factor 10 |
| ENSMUSG00000032554 | 1,074.3 | -1.29 | 1.02E-04 | Trf | transferrin |
| ENSMUSG00000085272 | 873.1 | -1.30 | 1.94E-02 | Sbk3 | SH3 domain binding kinase family, member 3 |
| ENSMUSG00000020120 | 452.4 | -1.31 | 1.50E-04 | Plek | pleckstrin |
| ENSMUSG00000026826 | 398.4 | -1.35 | 2.04E-02 | Nr4a2 | nuclear receptor subfamily 4, group A, member 2 |
| ENSMUSG00000037447 | 1,209.9 | -1.41 | 5.50E-11 | Arid5a | AT rich interactive domain 5A (MRF1-like) |
| ENSMUSG00000061451 | 167.1 | -1.41 | 1.27E-03 | Tmem151a | transmembrane protein 151A |
| ENSMUSG00000033730 | 470.2 | -1.41 | 3.21E-02 | Egr3 | early growth response 3 |
| ENSMUSG00000035000 | 284.5 | -1.45 | 6.64E-03 | Dpp4 | dipeptidylpeptidase 4 |
| ENSMUSG00000026580 | 212.9 | -1.46 | 1.44E-02 | Selp | selectin, platelet |
| ENSMUSG00000086320 | 336.5 | -1.50 | 1.49E-02 | Gm12840 | predicted gene 12840 |
| ENSMUSG00000064368 | 10,851.4 | -1.51 | 1.99E-04 | mt-Nd6 | mitochondrially encoded NADH dehydrogenase 6 |
| ENSMUSG00000031906 | 169.4 | -1.54 | 3.25E-03 | Smpd3 | sphingomyelin phosphodiesterase 3, neutral |
| ENSMUSG00000009281 | 811.4 | -1.57 | 1.62E-04 | Rarres2 | retinoic acid receptor responder (tazarotene induced) 2 |
| ENSMUSG00000041607 | 136.1 | -1.58 | 8.21E-03 | Mbp | myelin basic protein |
| ENSMUSG00000073418 | 692.7 | -1.63 | 2.96E-06 | C4b | complement component 4B (Chido blood group) |
| ENSMUSG00000024810 | 103.4 | -1.63 | 1.74E-02 | Il33 | interleukin 33 |
| ENSMUSG00000050335 | 142.7 | -1.66 | 3.74E-05 | Lgals3 | lectin, galactose binding, soluble 3 |
| ENSMUSG00000029869 | 87.2 | -1.77 | 2.04E-02 | Ephb6 | Eph receptor B6 |
| ENSMUSG00000038725 | 1,035.7 | -1.78 | 4.03E-05 | Pkhd1l1 | polycystic kidney and hepatic disease 1-like 1 |
| ENSMUSG00000013584 | 335.0 | -1.78 | 2.01E-03 | Aldh1a2 | aldehyde dehydrogenase family 1, subfamily A2 |
| ENSMUSG00000030772 | 1,198.4 | -1.83 | 6.07E-03 | Dkk3 | dickkopf WNT signaling pathway inhibitor 3 |
| ENSMUSG00000031574 | 54.4 | -1.83 | 8.24E-03 | Star | steroidogenic acute regulatory protein |
| ENSMUSG00000025746 | 260.4 | -1.91 | 1.03E-02 | Il6 | interleukin 6 |
| ENSMUSG00000034687 | 105.8 | -2.00 | 1.52E-04 | Fras1 | Fraser extracellular matrix complex subunit 1 |
| ENSMUSG00000024164 | 8,540.1 | -2.05 | 5.52E-10 | C3 | complement component 3 |
| ENSMUSG00000028341 | 1,297.6 | -2.08 | 2.10E-05 | Nr4a3 | nuclear receptor subfamily 4, group A, member 3 |
| ENSMUSG00000020473 | 1,177.4 | -2.11 | 1.85E-13 | Aebp1 | AE binding protein 1 |
| ENSMUSG00000001739 | 261.4 | -2.14 | 2.39E-05 | Cldn15 | claudin 15 |
| ENSMUSG00000034282 | 48.0 | -2.19 | 1.44E-02 | Evpl | envoplakin |
| ENSMUSG00000006403 | 274.9 | -2.22 | 8.55E-04 | Adamts4 | a disintegrin-like and metallopeptidase (reprolysin type) with thrombospondin type 1 motif, 4 |
| ENSMUSG00000053897 | 260.9 | -2.23 | 2.34E-07 | Slc39a8 | solute carrier family 39 (metal ion transporter), member 8 |
| ENSMUSG00000018849 | 51.5 | -2.24 | 3.77E-03 | Wwc1 | WW, C2 and coiled-coil domain containing 1 |
| ENSMUSG00000031517 | 523.2 | -2.25 | 1.80E-08 | Gpm6a | glycoprotein m6a |
| ENSMUSG00000055653 | 447.7 | -2.33 | 2.79E-11 | Gpc3 | glypican 3 |
| ENSMUSG00000000308 | 43.4 | -2.37 | 2.13E-02 | Ckmt1 | creatine kinase, mitochondrial 1, ubiquitous |
| ENSMUSG00000023046 | 932.1 | -2.43 | 1.10E-07 | Igfbp6 | insulin-like growth factor binding protein 6 |
| ENSMUSG00000004814 | 66.9 | -2.45 | 3.52E-05 | Ccl24 | chemokine (C-C motif) ligand 24 |
| ENSMUSG00000003665 | 136.7 | -2.49 | 3.64E-05 | Has1 | hyaluronan synthase 1 |
| ENSMUSG00000023043 | 119.0 | -2.51 | 4.37E-07 | Krt18 | keratin 18 |
| ENSMUSG00000001225 | 77.9 | -2.51 | 2.49E-02 | Slc26a3 | solute carrier family 26, member 3 |
| ENSMUSG00000027832 | 131.0 | -2.53 | 6.09E-03 | Ptx3 | pentraxin related gene |
| ENSMUSG00000020467 | 618.6 | -2.54 | 1.62E-11 | Efemp1 | epidermal growth factor-containing fibulin-like extracellular matrix protein 1 |
| ENSMUSG00000032068 | 35.8 | -2.55 | 1.91E-02 | Plet1 | placenta expressed transcript 1 |
| ENSMUSG00000031283 | 87.3 | -2.57 | 1.31E-04 | Chrdl1 | chordin-like 1 |
| ENSMUSG00000022523 | 154.5 | -2.60 | 1.28E-03 | Fgf12 | fibroblast growth factor 12 |
| ENSMUSG00000029082 | 71.3 | -2.69 | 1.40E-04 | Bst1 | bone marrow stromal cell antigen 1 |
| ENSMUSG00000090231 | 86.2 | -2.77 | 8.78E-03 | Cfb | complement factor B |
| ENSMUSG00000067276 | 48.1 | -2.79 | 1.13E-03 | Capn6 | calpain 6 |
| ENSMUSG00000037005 | 100.9 | -2.85 | 6.00E-03 | Xpnpep2 | X-prolyl aminopeptidase (aminopeptidase P) 2, membrane-bound |
| ENSMUSG00000026070 | 62.4 | -2.86 | 1.94E-02 | Il18r1 | interleukin 18 receptor 1 |
| ENSMUSG00000040612 | 155.8 | -2.87 | 4.31E-07 | Ildr2 | immunoglobulin-like domain containing receptor 2 |
| ENSMUSG00000044006 | 231.0 | -2.91 | 1.19E-06 | Cilp2 | cartilage intermediate layer protein 2 |
| ENSMUSG00000035403 | 51.8 | -3.00 | 7.16E-03 | Crb2 | crumbs family member 2 |
| ENSMUSG00000049382 | 111.6 | -3.03 | 3.54E-04 | Krt8 | keratin 8 |
| ENSMUSG00000026051 | 43.0 | -3.04 | 5.52E-04 | Ecrg4 | ECRG4 augurin precursor |
| ENSMUSG00000027015 | 143.1 | -3.05 | 4.50E-09 | Cybrd1 | cytochrome b reductase 1 |
| ENSMUSG00000028871 | 137.1 | -3.05 | 3.34E-06 | Rspo1 | R-spondin 1 |
| ENSMUSG00000054545 | 52.4 | -3.15 | 4.90E-06 | Ugt1a6a | UDP glucuronosyltransferase 1 family, polypeptide A6A |
| ENSMUSG00000041559 | 524.3 | -3.16 | 1.29E-13 | Fmod | fibromodulin |
| ENSMUSG00000035930 | 93.2 | -3.16 | 8.65E-05 | Chst4 | carbohydrate sulfotransferase 4 |
| ENSMUSG00000027574 | 147.5 | -3.30 | 1.84E-06 | Nkain4 | Na+/K+ transporting ATPase interacting 4 |
| ENSMUSG00000020911 | 178.3 | -3.42 | 2.80E-04 | Krt19 | keratin 19 |
| ENSMUSG00000040703 | 165.8 | -3.44 | 9.37E-07 | Cyp2s1 | cytochrome P450, family 2, subfamily s, polypeptide 1 |
| ENSMUSG00000063011 | 542.7 | -3.61 | 7.68E-03 | Msln | mesothelin |
| ENSMUSG00000025105 | 63.2 | -3.63 | 7.75E-06 | Bnc1 | basonuclin 1 |
| ENSMUSG00000024371 | 216.5 | -3.68 | 3.91E-11 | C2 | complement component 2 (within H-2S) |
| ENSMUSG00000109564 | 283.6 | -3.74 | 2.54E-02 | Muc16 | mucin 16 |
| ENSMUSG00000042985 | 1,283.4 | -3.76 | 2.52E-05 | Upk3b | uroplakin 3B |
| ENSMUSG00000027070 | 166.7 | -3.84 | 3.28E-07 | Lrp2 | low density lipoprotein receptor-related protein 2 |
| ENSMUSG00000049436 | 161.4 | -3.86 | 2.10E-05 | Upk1b | uroplakin 1B |
| ENSMUSG00000023039 | 119.4 | -3.86 | 1.30E-04 | Krt7 | keratin 7 |
| ENSMUSG00000043110 | 411.7 | -3.98 | 3.07E-11 | Lrrn4 | leucine rich repeat neuronal 4 |
| ENSMUSG00000027840 | 41.5 | -4.20 | 1.30E-04 | Wnt2b | wingless-type MMTV integration site family, member 2B |
| ENSMUSG00000060962 | 31.7 | -4.25 | 1.91E-03 | Dmkn | dermokine |
| ENSMUSG00000017002 | 16.0 | -4.58 | 2.83E-02 | Slpi | secretory leukocyte peptidase inhibitor |
| ENSMUSG00000057092 | 15.3 | -5.70 | 2.19E-02 | Fxyd3 | FXYD domain-containing ion transport regulator 3 |
| ENSMUSG00000078880 | 31.2 | -5.98 | 3.96E-04 | Gm14308 | predicted gene 14308 |
| ENSMUSG00000061780 | 45.7 | -6.27 | 6.68E-04 | Cfd | complement factor D (adipsin) |
| ENSMUSG00000027559 | 64.9 | -6.91 | 4.71E-05 | Car3 | carbonic anhydrase 3 |
| ENSMUSG00000098404 | 73.6 | -8.07 | 1.13E-02 | Mrip-ps | Mom radiation induced polyposis, pseudogene |
| ENSMUSG00000031362 | 1.9 | -14.48 | 7.94E-04 | Xlr4c | X-linked lymphocyte-regulated 4C |
| ENSMUSG00000094420 | 1.3 | -14.85 | 4.39E-02 | Igkv10-96 | immunoglobulin kappa variable 10-96 |
| ENSMUSG00000083813 | 0.6 | -15.25 | 3.15E-02 | Gm15502 | predicted gene 15502 |
| ENSMUSG00000117286 | 1.0 | -15.72 | 2.07E-02 | Gm1043 | predicted gene 1043 |
| ENSMUSG00000096336 | 1.6 | -15.77 | 1.94E-02 | Igkv1-135 | immunoglobulin kappa variable 1-135 |
| ENSMUSG00000096108 | 1.4 | -15.87 | 8.67E-03 | Ighv11-2 | immunoglobulin heavy variable V11-2 |
| ENSMUSG00000105906 | 2.0 | -15.91 | 2.27E-03 | Iglc1 | immunoglobulin lambda constant 1 |
| ENSMUSG00000092329 | 289.2 | -16.04 | 9.61E-03 | Galnt2l | polypeptide N-acetylgalactosaminyltransferase 2-like |
| ENSMUSG00000095589 | 1.7 | -16.47 | 1.03E-02 | Ighv1-55 | immunoglobulin heavy variable 1-55 |
| ENSMUSG00000093861 | 1.9 | -17.14 | 5.57E-03 | Igkv1-110 | immunoglobulin kappa variable 1-110 |
| ENSMUSG00000076934 | 3.9 | -17.31 | 2.18E-03 | Iglv1 | immunoglobulin lambda variable 1 |
| ENSMUSG00000076569 | 6.8 | -17.43 | 6.10E-08 | Igkv5-39 | immunoglobulin kappa variable 5-39 |
| ENSMUSG00000073234 | 0.8 | -18.34 | 1.96E-03 | Gm8773 | predicted gene 8773 |
| ENSMUSG00000042938 | 202.4 | -18.94 | 9.97E-04 | Gm14117 | predicted gene 14117 |
| ENSMUSG00000082585 | 153.2 | -19.15 | 6.76E-04 | Gm15387 | predicted gene 15387 |
| ENSMUSG00000076532 | 1.4 | -19.83 | 3.50E-04 | Igkv4-91 | immunoglobulin kappa chain variable 4-91 |
| ENSMUSG00000076613 | 7.6 | -22.41 | 1.95E-16 | Ighg2b | immunoglobulin heavy constant gamma 2B |
| ENSMUSG00000090381 | 63.8 | -23.82 | 2.70E-06 | Gm6158 | predicted gene 6158 |

**Supplemental Table 5.** Differentially expressed genes in CSP vs CTRL adjusted p ≤ 0.05), sorted by log2fold change CSP vs CTRL.

# Supplemental Table 6. PROGENy Pathway Analysis

| pathway | score | p value |
| --- | --- | --- |
| Androgen | -3.2 | 0.001 |
| EGFR | -0.9 | 0.377 |
| Estrogen | -0.2 | 0.822 |
| Hypoxia | 2.2 | 0.026 |
| JAK-STAT | 2.8 | 0.005 |
| MAPK | 1.3 | 0.183 |
| NFkB | -7.2 | 2.7E-12 |
| PI3K | 0.2 | 0.875 |
| TGFb | 0.4 | 0.655 |
| TNFa | 2.6 | 0.009 |
| Trail | -1.7 | 0.085 |
| VEGF | -1.2 | 0.221 |
| WNT | -1.9 | 0.059 |
| p53 | -0.8 | 0.432 |

**Supplemental Table 6.** Results from the PROGENy pathway analysis of CSP vs CTRL, using the top 1000 genes of each pathway of the mouse model.
